# A Bacterial mRNA‐Lysis‐Mediated Cargo Release Vaccine System for Regulated Cytosolic Surveillance and Optimized Antigen Delivery

**DOI:** 10.1002/advs.202303568

**Published:** 2023-10-22

**Authors:** Yu‐an Li, Yanni Sun, Yuqin Zhang, Quan Li, Shifeng Wang, Roy Curtiss, Huoying Shi

**Affiliations:** ^1^ College of Veterinary Medicine Yangzhou University Yangzhou Jiangsu 225000 China; ^2^ Jiangsu Co‐innovation Center for the Prevention and Control of Important Animal Infectious Diseases and Zoonoses Yangzhou 225000 China; ^3^ Department of Infectious Diseases and Immunology College of Veterinary Medicine University of Florida Gainesville FL 32611‐0880 USA; ^4^ Joint International Research Laboratory of Agriculture and Agri‐Product Safety Yangzhou University (JIRLAAPS) Yangzhou 225000 China

**Keywords:** cGAS‐STING, cytosolic surveillance, MazF interferase, Salmonella

## Abstract

Engineered vector‐based in vivo protein delivery platforms have made significant progress for both prophylactic and therapeutic applications. However, the lack of effective release strategies results in foreign cargo being trapped within the vector, restricting the provision of significant performance benefits and enhanced therapeutic results compared to traditional vaccines. Herein, the development of a Salmonella mRNA interferase regulation vector (SIRV) system is reported to overcome this challenge. The genetic circuits are engineered that (1) induce self‐lysis to release foreign antigens into target cells and (2) activate the cytosolic surveillance cGAS‐STING axis by releasing DNA into the cytoplasm. Delayed synthesis of the MazF interferase regulates differential mRNA cleavage, resulting in a 36‐fold increase in the delivery of foreign antigens and modest activation of the inflammasome, which collectively contribute to the marked maturation of antigen‐presenting cells (APCs). Bacteria delivering the protective antigen SaoA exhibits excellent immunogenicity and safety in mouse and pig models, significantly improving the survival rate of animals challenged with multiple serotypes of Streptococcus suis. Thus, the SIRV system enables the effective integration of various modular components and antigen cargos, allowing for the generation of an extensive range of intracellular protein delivery systems using multiple bacterial species in a highly efficient manner.

## Graphical abstract

The SIRV system enhances the safety and immunogenicity of *Salmonella* vectors through the following mechanisms: (1) induce self‐lysis to release foreign antigens; (2) activate the cytosolic surveillance cGAS‐STING axis; (3) reduce inflammasome activation by impairing flagellin production; (4) promote APC‐maturity, thus improving antigen presentation and immunoresponse elicitation.

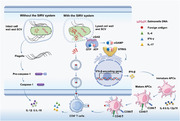

## 1 Introduction

The target repertoire for disease treatment and prevention can be expanded by delivering protein‐based drugs or antigens to the body. This has led to the emergence of various delivery techniques such as nanoparticles, cell‐penetrating peptides, and antibody‐drug conjugates. However, the effectiveness of these methods is limited by (1) poor cellular uptake, (2) lack of specificity for antigen‐presenting cells (APCs), and (3) inability to escape from intracellular compartments.^[^ ^1^, ^2^, ^3^, ^4^ ^]^

When particles and proteins are taken up by cells, they are trafficked from the early and late endosomes to the lysosomes, where they are degraded.^[^ ^4^, ^5^, ^6^ ^]^ The use of bacteria changes the traditional notion of “delivery.” Unlike conventional vectors, bacteria manufacture exogenous proteins in vivo,^[^ ^7^ ^]^ delivering more molecules than those present through injection. Numerous innovative projects are seeking to leverage bacterial vectors to develop prophylactic and therapeutic vaccines against infectious diseases and cancer.^[^ ^4^, ^8^, ^9^ ^]^

Due to their unique physiology, *Salmonella* vectors are particularly suitable for the delivery of protein drugs or antigens into the body. *Salmonella* (1) actively invades and colonizes the host lymphatic system, (2) constantly produces antigens or drug molecules to stimulate the lymphatic system, and (3) activates the innate immune system to provide adjuvant properties.^[^ ^10^, ^11^ ^]^ Generally, *Salmonella* delivery systems must include three basic components: (1) reasonable attenuation to ensure safety, (2) synthesis of exogenous proteins, and (3) adequate release.^[^ ^12^, ^13^ ^]^ However, previous *Salmonella* delivery vectors have been difficult to fully meet these requirements, especially with regard to foreign cargo trapped within the bacterial cytoplasm, which is difficult to release. This severely limits the widespread application of bacterial vectors.

MazF is an interferase from *Escherichia coli* that cleaves single‐stranded mRNA with a 5′‐ACA‐3′ sequence.^[^ ^14^ ^]^ This enzymatic property provides a strategy for targeted protein regulation: the gene encoding the target protein is designed to lack an ACA nucleotide sequence (ACA‐), whereas mRNA from other genes containing ACA sequences (ACA+) is degraded, resulting in a significant increase in the relative expression of the target protein.^[^ ^15^ ^]^ Interestingly, owing to codon diversity, any gene can be designed in an ACA‐free form without altering the amino acid sequence.^[^ ^15^ ^]^ MazF mediates membrane atypia and rupture under radiation stress in *Deinococcus radiota*.^[^ ^16^ ^]^ The ectopic production of MazF in *E. coli* leads to the generation of excessively elongated cells.^[^ ^17^ ^]^ Moreover, in *E. coli* infected with phages, MazF accelerates cell membrane damage.^[^ ^18^ ^]^ These studies suggest that MazF can mediate membrane damage in bacteria, indicating that it may be possible to use the regulation of MazF expression as a mechanism to release cargo from the constraints of the bacterial membrane system. MazF confers resistance to human immunodeficiency virus (HIV) on T cells in primates and humans without affecting normal cell growth,^[^ ^19^, ^20^ ^]^ demonstrating potential for in vivo applications.

*S. suis*, a widespread bacterial colonizer of pigs, has recently expanded its host range to humans, resulting in a spike in fatal human infections worldwide.^[^ ^21^ ^]^ Particularly, *S.suis* has emerged as the primary pathogen causing meningitis in certain Asian countries, thus posing a significant risk to public health.^[^ ^22^, ^23^, ^24^ ^]^ Although vaccination presents a promising targeted approach to combat the disease, the numerous serotypes present a challenge for the development of a universal vaccine.^[^ ^25^ ^]^ SaoA is a highly conserved *S.suis* antigen.^[^ ^26^ ^]^ We have previously reported that it can induce cross‐reactivity against different serotypes of *S. suis*.^[^ ^27^ ^]^

**Scheme 1:**
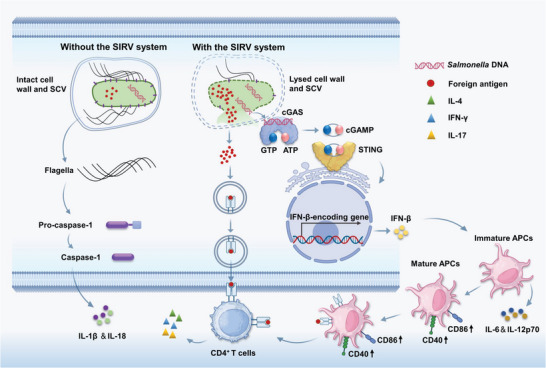
Diagram of model illustrating SIRV. The SIRV system enhances the safety and immunogenicity of *Salmonella* vectors through the following mechanisms: (1) induction of self‐lysis to release foreign antigens; (2) activation of the cytosolic surveillance cGAS‐STING axis; (3) reduction of inflammasome activation by impairing flagellin production; and (4) promotion of APC maturity, thus improving antigen presentation and immunoresponse elicitation.

We hypothesized that introducing a regulated MazF into the *Salmonella* vector would enable the delivering and release of large quantities of the antigen. Therefore, we developed a *Salmonella* mRNA interferase regulation vector (SIRV) system (**Scheme** **1**). Using SaoA as a model antigen, the SIRV system (1) induces massive production and active release of SaoA by differentially regulating mRNA degradation. (2) The released bacterial cytoplasmic inclusions enhance recognition by the host cytoplasmic surveillance pathway, promote APC maturation and the presentation of SaoA, (3) and strengthen adjuvant activity. Subsequent mouse and pig experiments showed strong preventive effects against multiple serotypes of *S. suis* infection. Therefore, our approach provides an attractive platform for the efficient synthesis and release of foreign antigenic cargo in vivo to produce a variety of high‐performance universal vaccines.

## 2 Results

### 2.1 Rationale and Construction of the SIRV System

**Figure 1:**
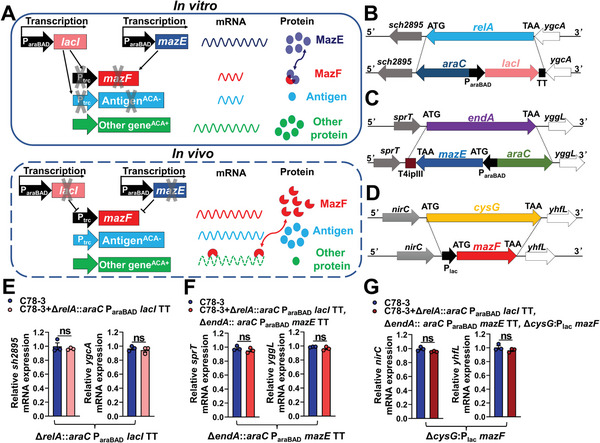
Design principle of SIRV. A) The mechanism of the SIRV system. Schematic map of Δ*relA*::*araC* P_araBAD_ *lacI* TT deletion‐insertion mutation B), Δ*endA*::*araC* P_araBAD_ *mazE* TT deletion‐insertion mutation C), and Δ*cysG*:P_lac_ *mazF* deletion‐insertion mutation D). The mRNA levels of the *sh2895* and *ygcA* genes adjacent to *relA* E), the *yggL* and *sprT* genes adjacent to *endA* F), and the *nirC* and *yhfL* genes adjacent to *cysG* G). E–G) *n* = three biological replicates per group. Data are expressed as the mean ± standard error of the mean (SEM). Adjusted *P* values were calculated by student's *t*‐test. Asterisks indicate significant differences between groups linked by horizontal lines. ns, not significant.

The SIRV system comprises three chromosomal mutations: *araC* P_araBAD_ *lacI* TT, *araC* P_araBAD_ *mazE* TT, P_lac_ *mazF* deletion‐insertion mutation, and a plasmid carrying a P_trc_ regulated ACA antigen gene for the regulated delayed synthesis of antigen and MazF. The production of the interferase MazF can cause cell growth arrest, lysis, and death.^[^ ^28^, ^29^ ^]^ To minimize the detrimental effects of MazF in vitro. The LacI‐repressible promoter P_lac_ regulates *mazF* expression at the mRNA level^[^ ^30^ ^]^ whereas the leaky production of MazF can be further neutralized by the antitoxin MazE at the protein level.^[^ ^29^ ^]^ The *araC* P_araBAD_ promoter regulates *mazE* and *lacI* expression, generating a dual shut‐off mechanism at both mRNA and protein levels, which minimizes the toxicity of MazF in vitro. Transcription of *mazF*, ACA‐ antigen gene, and ACA+ genes commenced. The simultaneous occurrence of antigen synthesis and cleavage of ACA+ mRNAs by MazF causes the antigen to become the dominant protein produced in vivo, inducing immune responses (**Figure** **1**A).

**Figure 2:**
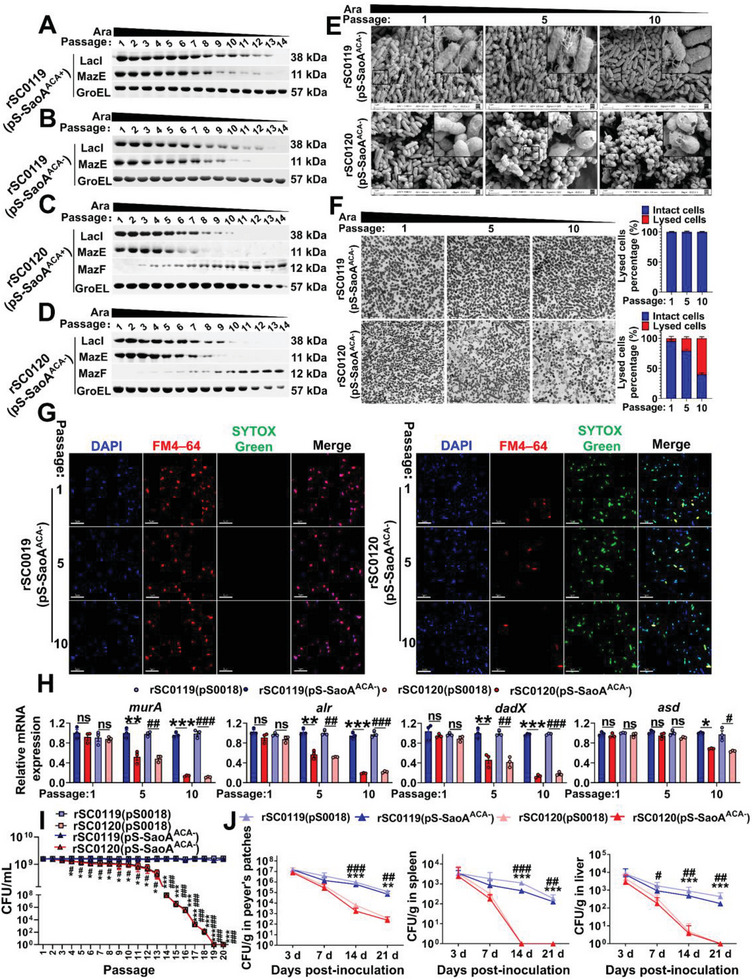
SIRV alters cell morphology and gene expression, causing the strain to self‐lyse. rSC0119 or rSC0120 carrying pS‐SaoA^ACA+^ A,C) or pS‐SaoA^ACA−^ B,D) were serially passaged in arabinose‐free medium supplemented with 0.2% (w/v) arabinose during the primary generation. Arabinose regulates the synthesis of LacI, MazE, and MazF, which were analyzed using western blotting. E) SEM analysis of rSC0119(pS‐SaoA^ACA−^) and rSC0120(pS‐SaoA^ACA−^) at passages 1, 5, and 10. Scale bars, 1 µm. F) TEM analysis of rSC0119(pS‐SaoA^ACA−^) and rSC0120(pS‐SaoA^ACA−^) at passages 1, 5, and 10. Scale bars, 5 µm (left). Quantification of lysed cells from 200 individual bacterial cells per strain (right). G) Fluorescence analysis of rSC0119(pS‐SaoA^ACA−^) and rSC0120(pS‐SaoA^ACA−^) at passages 1, 5, and 10. DAPI, FM4‐64, and SYTOX Green‐only images, or overlay (FM4‐64, DAPI, and SYTOX Green) images are shown. Scale bars, 5 µm. H) mRNA levels of genes involved in bacterial cell wall peptidoglycan synthesis at passages 1, 5, and 10. I) The indicated strains were serially passaged in arabinose‐free medium supplemented with 0.2% (w/v) arabinose during the primary generation. The bacterial density was measured. J) Colonization of mice on days 3, 7, 14, and 21 with the indicated strains after oral inoculation with 10^9^ CFU bacteria. Ten CFU per Peyer's patch or gram of tissue was set as the assay's limits of detection. A–G) *n* = three biological replicates per group; a representative sample is shown. H,I) *n* = three biological replicates per group. J) *n* Five mice. H–J) Data are expressed as mean ± SEM. Adjusted *P* values were calculated by one‐way ANOVA with Tukey's multiple comparison test. Asterisks indicate significant differences between groups linked by horizontal lines. ns, not significant; ^###^ *P* < 0.001, ^##^ *P* < 0.01, ^#^ *P* < 0.05, compared with strains rSC0119(pS0018) and rSC0120(pS0018); ***P < 0.001, **P < 0.01, *P < 0.05, compared with strains rSC0119(pS‐SaoA^ACA−^) and rSC0120(pS‐SaoA^ACA−^).

The cassettes *araC* P_araBAD_ *lacI* TT, *araC* P_araBAD_ *mazE* TT, and P_lac_ *mazF* were introduced into *relA*, *endA*, and *cysG*, respectively (Figure 1B–D). Transcription terminators (TT) at the C terminals of *mazE* and *lacI* preclude *mazF* or *lacI* transcription reading through adjacent genes and interfere with the function of adjacent genes. Reverse transcription‐polymerase chain reaction (RT‐PCR) analysis revealed that the expression of the adjacent genes did not change (Figure 1E–G). rSC0120 is a SIRV strain, whereas rSC0119 is a non‐SIRV control as it lacks the mutation Δ*cysG*::P_lac_ *mazF*. Owing to the regulatory effect of arabinose on the SIRV system, the synthesis of LacI and MazE in rSC0119 and rSC0120 occurred at an early stage, with termination at later stages of passage (**Figure** **2**A–D). In addition, MazF production increased (Figure 2A–D).

To prevent MazF‐mediated cleavage of foreign antigens, the *asdA* gene in control plasmid pS0018 was engineered as an ACA (Figure S1A, Supporting Information). The *saoA* gene from *S. suis* serotype 2 (SS2), with and without ACA base triplets, was cloned into plasmid pS0018 to generate plasmids pS‐SaoA^ACA‐^ and pS‐SaoA^ACA+^, respectively (Figure S1A, Supporting Information). The stable maintenance of plasmids is essential to ensure the efficacy of RASV. Plasmid pS‐SaoA^ACA−^ is stable in both rSC0119 and pSC0120 in non‐selective media for 50 generations, indicating that the SIRV system did not affect plasmid stability (Figure S1C, Supporting Information) or SaoA production (Figure S1D).

### 2.2 SIRV Mediates Programmed Self‐Lysis In Vitro and In Vivo

MazF has been reported to alter bacterial cell morphology.^[^ ^16^, ^17^ ^]^ Consistent with this, the presence of tunnel‐like structures in the cell wall and plasma membrane of strain rSC0120(pS‐SaoA^ACA−^) at passages 5th and 10th passages were observed using transmission electrone microscopy (TEM) and scanning electron microscopy (SEM) analyses (Figure 2E,F). The strain exhibited a change in cell shape from rod to round and eventually collapsed, leading to leakage of cytoplasmic content. In contrast, no changes in cell morphology were observed in rSC0119(pS‐SaoA^ACA−^) cells (Figure 2E; Figure S2A, Supporting Information). The proportion of lysed cells and cells with membrane tunnels increased with the enhanced production of MazF in rSC0120(pS‐SaoA^ACA−^), with >60% of cells lysed by the 10th passage, but not in strain rSC0119(pS‐SaoA^ACA−^) (Figure 2F). The non‐SIRV strain rSC0119 was stained with the lipophilic membrane dye FM4‐64 to produce red fluorescence, but not with the impermeable nuclear dye SYTOX‐green (Figure 2G). In contrast, the phospholipid layer of the SIRV strain rSC0120 did not bind to FM4‐64, whereas its nuclei were stained with SYTOX‐green through the damaged cell wall and plasma membrane (Figure 2G). These results suggest that MazF damages the phospholipid layer of SIRV, leading to the formation of membrane channels that allow the entry of impermeable nuclear staining dyes into *Salmonella*. Moreover, we observed a gradual reduction in the transcription levels of the cell wall synthesis genes *murA*, *alr*, *dadX*, and *asd* during passaging (Figure 2H), with 11, 11, 12, and 10 ACA triplets, respectively (Table S2, Supporting Information). A *Salmonella* intrinsic cytoplasmic marker α‐galactosidase (α‐gal) only exists in the cytoplasm of strain rSC0119, but gradually reduces in the cytoplasm and increases in the periplasm and supernatant of rSC0120 with the passages (Figure S2B, Supporting Information). The foreign β‐galactosidase (β‐gal) and SaoA, which lack a signal peptide, can be released into the periplasm and supernatant by SRIV strain rSC0120 during continuous passaging, but not rSC0119 (Figure S2C–E, Supporting Information). Both chromosomal and plasmid DNA were detected in the periplasm and supernatant of rSC0120 but not in rSC0119, despite no differences in the cytoplasm (Figure S2F,G, Supporting Information). Collectively, our findings indicate the superior ability of the SIRV strain to produce and release foreign antigens. The release process occurred in a signal peptide‐independent manner.

The SIRV strain rSC0120 exhibited an arabinose‐regulated delayed death phenotype, as evidenced by a gradual decrease in colony numbers during passages in arabinose‐free media (Figure 2I). In contrast, the growth of the rSC0119 cells was unaffected (Figure 2I). The LD_50_ of the intraperitoneal injection of strain rSC0120(pS‐SaoA^ACA−^) was 17‐fold higher than that of rSC0119(pS‐SaoA^ACA−^), although both were safe for oral immunization (Table S3, Supporting Information). SIRV strain rSC0120 with either plasmid was cleared in the spleen on day 14 and in the liver on day 21 after inoculation (Figure 2J); however, rSC0119 with either plasmid was still present on day 21. Notably, the colonization of rSC0119 and rSC0120 with either plasmid was similar before day 7. In addition, there were no significant differences in the colonization of rSC0120(pS0018) and rSC0120(pS‐SaoA^ACA−^) in the Peyer's patches, spleen, and liver, suggesting that the expression of foreign antigens did not affect the colonization of rSC0120(pS‐SaoA^ACA−^) (Figure 2J). These results suggest that rSC0120 can colonize the host lymphatic system at a level similar to that of rSC0119 in the early stages and undergo faster reduction than rSC0119 thereafter, ensuring safety and biocontainment at later stages.

### 2.3 SIRV Enhances Activation of the cGAS‐STING Pathway

**Figure 3:**
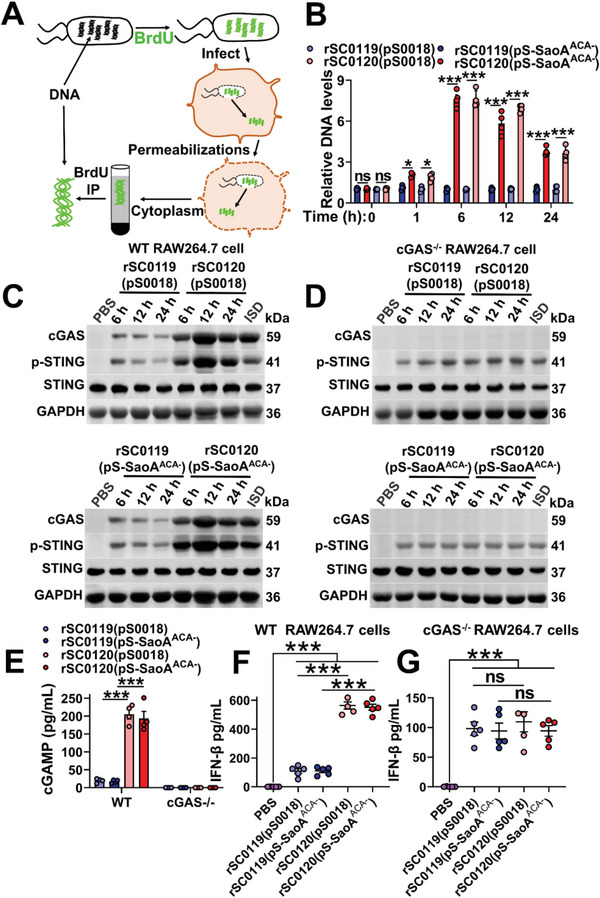
SIRV enhanced the activation of the cGAS‐STING axis. A) Strains rSC0119 and rSC0120, either with pS‐SaoA^ACA−^ or pS0018, were labeled with BrdU prior to RAW264.7 cell infection. Cell cytosolic fractions were separated 0, 1, 6, 12, and 24 h following infection, and BrdU‐labeled bacterial DNA was retrieved using immunoprecipitation with BrdU‐specific antibodies. B) Lysates from (A) were used as templates for qRT‐PCR quantification of *S*. Choleraesuis DNA. At 0, 1, 6, 12, and 24 h post‐infection with indicated strains, cGAS and p‐STING expression in WT RAW264.7 cells C) and cGAS KO RAW264.7 cells D) were assessed using western blotting. For all western blot analyses, GAPDH was used as a loading control. E) Analysis of catalytic activity of cGAS in RAW264.7 cells infected with the relevant strains. cGAMP in cell extracts was separated using chromatography with a C18 column, and cGAMP was quantitated using LC‐MS analysis. Secretory IFN‐β levels in were assessed 12 h post‐infection in WT RAW264.7 cells F), and cGAS KO RAW264.7 cells G) using ELISA. B) *n* = four biological replicates per group. C,D) *n* = three biological replicates per group; a representative sample is showed. E) *n* = four biological replicates per group. F,G) *n* = five biological replicates per group. B, E–G) Data Are expressed as the mean ± SEM. Adjusted *P* values were calculated by one‐way ANOVA with Tukey's multiple comparison test. Asterisks indicate significant differences between groups linked by horizontal lines. ns, not significant; ***, *P* < 0.001; **, *P* < 0.01.

Microbiota‐derived DNA is present within the cytoplasm and triggers tonic activation of the cytosolic cyclic GMP‐AMP synthase (cGAS)‐stimulator of interferon gene (STING) axis.^[^ ^31^ ^]^ Since the EM results showed that the SIRV strain rSC0120 released cytoplasmic contents through the pores in the cell membrane (Figure 2E,F), we further investigated whether bacterial‐derived DNA was released and activated the cGAS‐STING axis in response to SIRV. To detect the presence of the released bacterial‐derived DNA inside infected cells, cytoplasmic fractions were isolated from RAW264.7 cells infected with BrdU‐labeled strains rSC0119 and rSC0120 (**Figure** **3**A). *Salmonella* DNA was enriched in the cytoplasm of cells infected with the SIRV strain rSC0120, but not rSC0119, with a peak at 6 h (Figure 3B). These findings demonstrated that SIRV enables the release of *Salmonella* DNA into the cytoplasm of infected cells. The levels of cGAS and p‐STING in RAW264.7 cells infected with strain rSC0120 were significantly higher than those of rSC0119 (Figure 3C), with the largest difference observed at 6 h, which matched the peak DNA contents. Activation of cGAS and STING was not observed in cGAS KO RAW264.7 cells (Figure 3D). The levels of cGAMP were significantly higher in RAW264.7 cells infected with rSC0120 compare with rSC0119, whereas no cGAMP production was observed in cGAS KO RAW264.7 cells (Figure 3E). These data suggested that SIRV promoted STING activation by enhancing cGAS enzymatic activity. The secretion of IFN‐β by WT RAW264.7 cells infected with rSC0120 with either plasmid were significantly higher than those with rSC0119 with either plasmid (Figure 3F). The production of IFN‐β interferon in response to rSC0120 with either plasmid was dependent on cGAS signalling since cGAS KO cells failed to induce high levels of IFN‐β expression (Figure 3G). Similar results were observed for pig macrophages. SIRV strains rSC0120 with either plasmid activated higher levels of cGAS, p‐STING, cGAMP, and IFN‐β than those of rSC0119 with either plasmid did (Figure S3A–E, Supporting Information). Furthermore, we observed significantly higher concentrations of IFN‐β in serum in both pigs and mice after immunization with rSC0120 than with rSC0119 (Figure S3F,G, Supporting Information). Collectively, these data demonstrate that strain rSC0120 with the SIRV system is an agonist of the cGAS‐STING pathway in both pig‐ and mouse‐derived cells and is translated into the upregulated expression of type I interferon in vivo.

### 2.4 SIRV Enhanced Foreign Antigen Release and DC Maturation

**Figure 4:**
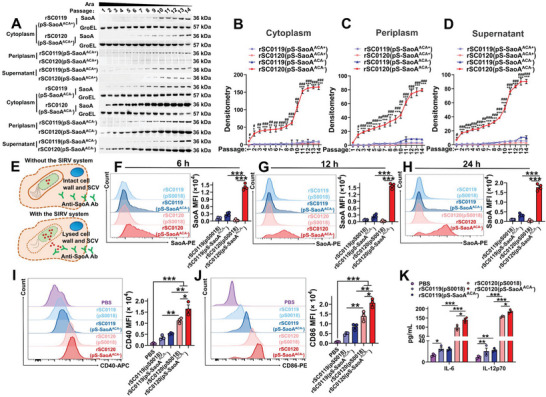
SIRV induces maturation of BMDCs and release of foreign antigens. rSC0119 or rSC0120 carrying pS‐SaoA^ACA+^ or pS‐SaoA^ACA−^ were serially passaged in arabinose‐free medium supplemented with 0.2% (w/v) arabinose during the primary generation. Arabinose regulates the synthesis of SaoA in cytoplasm, periplasm, and supernatant, which were analyzed using western blotting with an SaoA‐specific antibody and normalized to groEL; A) a representative image and densitometric measurements of SaoA in the cytoplasm B), periplasm C), and supernatant D) are shown. E) Selective permeabilization of BMDCs membranes enabled the detection of released SaoA at 6 F), 12 G), and 24 h H) post‐infection of BMDC with strains rSC0119 and rSC0120, either with pS‐SaoA^ACA−^ or pS0018. The mean fluorescence intensity (MFI) of SaoA in the cytoplasm of BMDCs was quantified using flow cytometry. Representative images are shown on the left, and the statistical analysis histograms are shown on the right. BMDCs were treated with PBS, rSC0119(pS0018), rSC0119(pS‐SaoA^ACA−^), rSC0120(pS0018), or rSC0120(pS‐SaoA^ACA−^). The MFI of CD40 I) and CD86 J) in these BMDCs were quantified using flow cytometry. Representative images are shown on the left, and the statistical analysis histograms are shown on the right. K) Levels of cytokines IL‐6 and IL‐12p70 in BMDCs supernatants; only cytokines with detectable levels are shown. B–D, I–K) *n* = three biological replicates per group. F–H) *n* = four biological replicates per group. B–D) Data are expressed as the mean ± SEM. ^###^ *P* < 0.001, ^##^ *P* < 0.01, ^#^ *P* < 0.05, for comparison with strains rSC0120(pS‐SaoA^ACA+^) and rSC0120(pS‐SaoA^ACA−^). ***P < 0.001, **P < 0.01, *P < 0.05, for comparison with strains rSC0119(pS‐SaoA^ACA−^) and rSC0120(pS‐SaoA^ACA−^). F–K) Data are expressed as the mean ± SEM. Adjusted *P* values were calculated by one‐way ANOVA with Tukey's multiple comparison test. Asterisks indicate significant differences between groups linked by horizontal lines. ***P < 0.001, **P < 0.01, *P < 0.05.

Gene expression regulation in *Salmonella* vaccines can be achieved by arabinose administration.^[^ ^32^ ^]^ To assess the production of foreign antigens in SIRV, we examined the expression levels and subcellular localization of the foreign antigen SaoA over multiple passages. Notably, arabinose was added only during the first passage and was subsequently excluded from the subculture medium. This deliberate approach aimed to mimic the in vivo growth environment devoid of arabinose. The arabinose concentration in each passage was further determined in both the culture medium and bacterial cells using high‐performance liquid chromatograohy (HPLC), with a detection limit of 5 ng mL^−1^. The results (Figure S2H, Supporting Information) revealed that arabinose was present in the first‐generation culture medium. The concentration fell below the detection limit in subsequent generations of the culture medium. Interestingly, arabinose remained detectable in the bacterial cells up to the 3rd generation. Thus, in the SRIV system, arabinose concentration was rapidly reduced in the cells. Induction of the SIRV system significantly increased SaoA synthesis and reduced the expression of background cellular proteins in rSC0120(pS‐SaoA^ACA−^). In contrast, SaoA production in strains rSC0119(pS‐SaoA^ACA+^), rSC0119(pS‐SaoA^ACA−^), and rSC0120(pS‐SaoA^ACA+^) was barely visible, with a reduction in arabinose concentration (Figure S4A–C, Supporting Information). SaoA was visible in the 2nd passage and gradually became the dominant cellular protein (Figure S4D, Supporting Information). Moreover, the synthesis of SaoA in rSC0120(pS‐SaoA^ACA−^) was significantly higher than that in the rSC0119(pS‐SaoA^ACA−^), rSC0119(pS‐SaoA^ACA+^), and rSC0120(pS‐SaoA^ACA+^) strains, in the cytoplasm, periplasm, and supernatant (**Figure** **4**A–D; Figure S4A–D, Supporting Information). At the 12th passage, SaoA production in rSC0120(pS‐SaoA^ACA−^) reached a peak and was 36‐fold higher than that in rSC0119(pS‐SaoA^ACA−^) in the cytoplasm (Figure 4A–D). Notably, the release of SaoA from rSC0120(pS‐SaoA^ACA−^) was significantly higher than that from rSC0119(pS‐SaoA^ACA−^) in the periplasm and supernatant (Figure 4A–D).

To explore the interaction between SIRV system mediated foreign antigens and APCs, we further characterized the SaoA production in bone marrow‐derived dendritic cells (BMDCs). No SaoA signal was detected in the cytoplasm of BMDCs infected with control vectors rSC0119(pS0018) and rSC0120(pS0018). SaoA production in DCs infected with rSC0120(pS‐SaoA^ACA−^) was significantly higher than those with rSC0119(pS‐SaoA^ACA+^) at different time points (Figure 4E–H), suggesting that SIRV mediates the release of more antigens into the cytoplasm of APCs. Two layers of membranes in *Salmonella* limit contact between the synthesized antigens and the cytoplasm. One is the bacterial plasma membrane and cell wall^[^ ^4^ ^]^ and the other is the *Salmonella*‐containing vacuole (SCV).^[^ ^33^ ^]^ The expression of *sifA* directly determines SCV biogenesis^[^ ^34^ ^]^ We showed that SIRV enables lysis and pore formation in bacterial membranes and cell walls. The detection of SaoA in the cytoplasm also suggested that the SCV membrane had ruptured, as evidenced by the gradual reduced mRNA levels of *sifA* in cells infected with rSC0120 (Figure S4E, Supporting Information). This observation can be explained by the fact that *sifA* contains 17 ACA triplets (Table S2, Supporting Information). These data suggest that the SIRV system cleaves the transcripts of *sifA* in addition to the cell wall synthesis genes, leading to the breakdown of SCV and facilitating the release of antigens into the cytoplasm.

The activation of the cGAS‐STING axis promotes APC maturation and leads to greater antigen processing and presentation.^[^ ^35^ ^]^ Therefore, we explored whether the SIRV system could promote APC maturation. The levels of the co‐stimulatory molecules CD40 and CD86 on the plasma membranes of BMDCs treated with rSC0120(pS0018) or rSC0120(pS‐SaoA^ACA−^) were significantly higher than those treated with rSC0119(pS0018) or rSC0119(pS‐SaoA^ACA−^) (Figure 4I,J), suggesting that strains with SIRV system are more potent in inducing APC maturation than those without the system. SaoA may exhibit weak immunostimulatory activity, as shown by the slight increase in CD40/CD86 levels observed in strain rSC0119(pS‐SaoA^ACA−^) compared to the same strain with the control vector pS0018, which did not reach a significant level (Figure 4I,J; Figure S5A,B, Supporting Information). This effect appeared to be enhanced by the presence of the SIRV system in BMDCs (Figure 4I,J), but not in bone marrow‐derived macrophages (BMDMs) (Figure S5A,B, Supporting Information). Foreign antigens escaping the endosomal system can result in specific cross‐presentation, leading to more active processing and presentation by APCs.^[^ ^36^ ^]^ The levels of the co‐stimulatory molecules CD40 and CD86 in BMDCs infected with rSC0120(pS‐SaoA^ACA−^) were significantly higher than in those infected with rSC0120(pS0018) (Figure 4I,J), but not in BMDMs (Figure S5A,B, Supporting Information). These observations suggest that the SIRV system may improve APC activity by promoting the release of foreign antigens into the cytosol and potential SCV escape effects. Further analysis of pro‐inflammatory cytokines showed that the SIRV strain rSC0120 with either pS‐SaoA^ACA−^ or pS0018 significantly increased the secretion of IL‐6 and IL‐12p70 compared to rSC0119 with either pS‐SaoA^ACA−^ or pS0018 in BMDCs (Figure 4K). Strain rSC0120(pS‐SaoA^ACA−^) induced higher levels of IL‐6 and IL‐12p70 than rSC0120(pS0018) (Figure 4K). Similar phenotypes were observed in BMDMs. (Figure S5, Supporting Information). Collectively, our findings demonstrate that the SIRV strain rSC0120 produces and releases more antigens, increases antigen presentation through the lysis of bacterial and SCV membranes, and promotes APCs maturation, which is conducive for mounting a strong adaptive immune response.

### 2.5 SIRV Actives a Moderate Inflammation by Reducing the Synthesis of Flagellin

**Figure 5:**
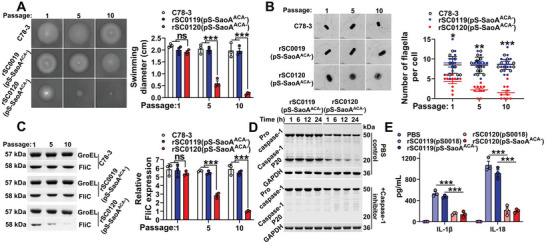
SIRV reduces inflammation. A) SIRV system affects swimming motility in strains rSC0119(pS‐SaoA^ACA−^) and rSC0120(pS‐SaoA^ACA−^). B) TEM analysis of strains rSC0119(pS‐SaoA^ACA−^) and rSC0120(pS‐SaoA^ACA−^). Scale bars, 1 µm (left). Measurement of surface flagella from ten distinct bacteria (right). C) FliC protein levels. Lysates from rSC0119(pS‐SaoA^ACA−^) and rSC0120(pS‐SaoA^ACA−^) were evaluated using western blotting; GroEL was used as a loading control D) Compared to rSC0119(pS‐SaoA^ACA−^), rSC0120(pS‐SaoA^ACA−^) decreases Caspase‐1 secretion (by ≈50 kDa) and proteolytic cleavage (by ≈20 kDa) during BMDC infection. Caspase‐1 was measured using western blotting with culture supernatants of rSC0119(pS‐SaoA^ACA−^)‐ or rSC0120(pS‐SaoA^ACA−^)‐infected BMDCs treated with or without the Caspase‐1 inhibitor; a GAPDH was used as a loading control. E) Levels of IL‐1β and IL‐18 were meaqsured in the culture supernatants of infected BMDCs using ELISA. A,C,E) *n* = three biological replicates per group; a representative sample is showed. B) Data are expressed as the mean ± SEM. Adjusted *P* values were calculated by one‐way ANOVA with Tukey's multiple comparison test. Asterisks indicate significant differences between groups linked by horizontal lines. ns, not significant; ***,*P* < 0.001; *, *P* < 0.05.

Flagella are the main pathogen‐associated molecular patterns (PAMP) on the surface of *Salmonella* and are closely associated with immunoregulation and inflammatory properties.^[^ ^37^ ^]^ Given the observed changes in the surface structure of the rSC0120 strains, we explored the phenotype of flagella under the action of the SIRV system. The swimming motility of rSC0120(pS‐SaoA^ACA−^) decreased, whereas that of rSC0119(pS‐SaoA^ACA−^) decreased (**Figure** **5**A). These phenotypes were consistent with a significant decrease in the number of peritrichous flagella per cell in rSC0120(pS‐SaoA^ACA−^) (Figure 5B). These observations were supported by concomitant changes in total FliC levels in these two strains (Figure 5C). *Salmonella* Choleraesuis has six flagellin genes totaling 109 trinucleotides (Table S2, Supporting Information), whereas other genes associated with flagellar assembly have varying numbers of ACA triplets. Decreased arabinose availability leads to increased MazF production. Consequently, MazF targets both *fliC* and other key genes involved in the flagellar system, resulting in its cleavage. Additionally, MazF leads to membrane rupture, further exacerbating flagellar reduction. As a cumulative effect, these processes collectively led to a decrease in flagella.

Flagellin induces NLRC4 inflammasome‐dependent activation of Caspase‐1 cell divisions, leading to the release of IL‐1β, IL‐18, and eventual pyroptosis in cells and causing tissue injury.^[^ ^38^ ^]^ Infection with rSC0119(pS‐SaoA^ACA−^) triggered Caspase‐1 processing, enriching the activation of the 20 kDa subunit (Figure 5D). This effect was abolished since no P20 was detected with the Caspase‐1 inhibitor (Figure 5D). Strain rSC0119 also induced the elevated secretion of IL‐1β and IL‐18 in BMDC (Figure 5E). By contrast, the SIRV strain rSC0120(pS‐SaoA^ACA−^) leads to reduced production of Caspase‐1, IL‐1β, and IL‐18 (Figure 5D,E).

### 2.6 SIRV Induces Comprehensive Improvements in Cellular, Humoral, and Mucosal Immunity to Foreign Antigen in Mice and Pigs

**Figure 6:**
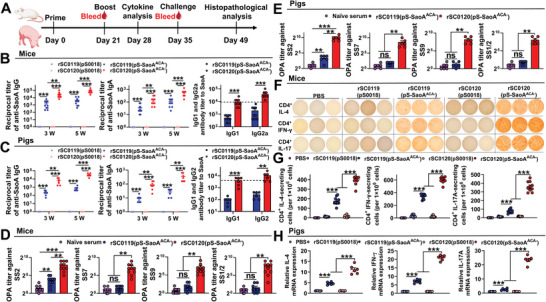
SIRV induces comprehensive improvements in cellular, humoral, and mucosal immunity in mice and pigs. A) Scheme of the immunization regimen. Serum IgG and vaginal wash IgA, IgG1, and IgG2a responses to SaoA in mice B) or pigs C) immunized with rSC0119(pS0018), rSC0119(pS‐SaoA^ACA−^), rSC0120(pS0018), or rSC0120(pS‐SaoA^ACA−^) were measured using ELISA. Bacterial killing by murine neutrophils under opsonizing conditions. SS2, SS7, SS9, and SS1/2 were incubated at 37 °C with rSC0119(pS‐SaoA^ACA−^)‐ and rSC0120(pS‐SaoA^ACA−^)‐immunized mouse D) or pig E) serum, then with porcine PMNs at a 1:1 (CFU:PMN) ratio for 1 h; OPA titer was determined as described in the Experimental section. ELISPOT assay of SaoA‐specific secreted cytokines from mice immunized with the relevant strains Representative ELISPOT plate F) showing IL‐4‐, IFN‐γ‐, and IL‐17‐producing colonies of CD4^+^ T cells stimulated with the SaoA and the mean number of spot forming units were determined G). H) SaoA‐specific cytokines from pigs immunized with the relevant strains as determined using qRT‐PCR. Pig peripheral blood mononuclear cells (PBMCs) were activated by SaoA, and relative mRNA expression levels of IL‐4, IFN‐γ, and IL‐17A were determined. A–F) *n* = ten mice or six pigs. G) *n* = four pigs. A–G) Data are expressed as mean ± SEM. Adjusted *P* values were calculated by one‐way ANOVA with Tukey's multiple comparison test. Asterisks indicate significant differences between groups linked by horizontal lines. ns, not significant; ***P < 0.001, **P < 0.01, *P < 0.05.

To assess the adaptive immunogenicity of SIRV, we explored cellular, humoral, and mucosal immune responses specific to the foreign antigen SaoA induced by the SIRV vector in mice and pigs (**Figure** **6**A). Body weight and clinical symptoms in mice and piglets after oral immunization with *Salmonella* strains were measured. No weight loss or adverse clinical symptoms were observed in any of the animals (data not shown). Serum IgG, IgG1, IgG2a (IgG2), and mucosal IgA antibodies induced by rSC0120(pS‐SaoA^ACA−^) were significantly higher than those induced by rSC0119(pS‐SaoA^ACA−^) in both mice and pigs (Figure 6B,C), indicating that SIRV delivering foreign antigens induced higher levels of humoral and mucosal immune responses. Functional opsonophagocytic (OPA) antibodies can better reflect antibody‐mediated immunity against *S*. *suis* than quantitative measurements.^[^ ^25^ ^]^ Although rSC0119(pS‐SaoA^ACA−^) induced significantly higher OPA responses against the homologous serotype SS2 than against naïve serum in mice and pigs (Figure 6D,E), it did not induce OPA responses against heterologous *S. suis* serotypes 7, 9, and 1/2 (SS7, SS9, and SS1/2) (Figure 6D,E). In contrast, rSC0120(pS‐SaoA^ACA−^) induced OPA antibodies against the homologous serotype SS2 and OPA responses against the heterologous serotypes SS7, SS9, and SS1/2 in mice and pigs (Figure 6D,E). These results suggested that SIRV mediates broad functional antibody responses in both homologous and heterologous *S. suis*. This could be a benefit of the increased antigen presentation to the immune system. IgG2a has an advantage over IgG1 in inducing OPA responses due to its higher affinity for the Fc receptor (FcR).^[^ ^39^ ^]^ Therefore, we hypothesized that the IgG2a‐dominant antibody response induced by SIRV translates into a more potent OPA response.

More SaoA‐specific CD4^+^/IFN‐γ^+^, CD4^+^/IL‐4, and CD4^+^/IL‐17A‐secreting cells were elicited in mice immunized with rSC0120(pS‐SaoA^ACA−^) than those with rSC0119(pS‐SaoA^ACA−^) (Figure 6F,G). This phenomenon has also been observed in pigs. The mRNA expression levels of IL‐4, IFN‐γ, and IL‐17A in pig splenocytes stimulated with SaoA from pigs immunized with rSC0120(pS‐SaoA^ACA−^) were significantly higher than those with rSC0119(pS‐SaoA^ACA−^) (Figure 6H). Both rSC0119(pS‐SaoA^ACA−^) and rSC0120(pS‐SaoA^ACA−^) induce higher IFN‐γ‐responses, indicating antigen‐specific Th1‐biased immune responses in both mice and pigs (Figure 6G,H). These data demonstrate that SIRV mediates the robust proliferation and differentiation of antigen‐specific T cells. Collectively, these findings demonstrate that SIRV mediates robust foreign antigen‐specific adaptive immune responses in both mouse and pig models.

### 2.7 SIRV Confers Protection against Homologous or Heterologous *S. Suis* Challenge

**Figure 7:**
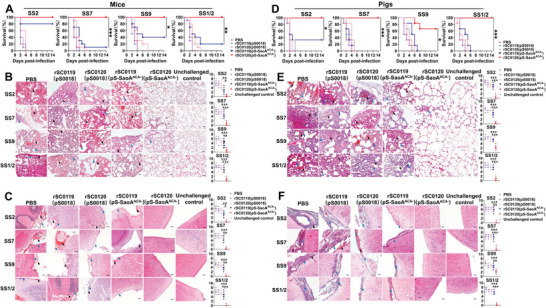
SIRV induces protection in mice and pigs. A) Survival curves, histopathologies of the lungs B) and the brains C) of immunized mice challenged i.p. with SS2, SS7, SS9, or SS1/2. D) Survival curves, histopathologies of the lungs E) and the brains F) of immunized pigs challenged i.v. with SS2, SS7, SS9, or SS1/2. The log‐rank Mantel‐Cox methodology for curve comparison analysis was used to perform the Kaplan‐Meier life survival curve analysis. For lung histopathology analysis, alveolar hyperemia is shown with a black arrow, and neutrophilic infiltration and interstitial thickening are shown with a blue arrow. For brain histopathology analysis, meningeal hemorrhaging and meningeal thickening are shown with a black arrow infiltration, and neutrophilic is shown with a blue arrow infiltration. B,C,E, and F) A representative histopathology image is shown on the left, and a histology score statistical analysis histogram is shown on the right. A–F) Data are expressed as the mean ± SEM. Adjusted *P* values were calculated by one‐way ANOVA with Tukey's multiple comparison test. Asterisks indicate significant differences between groups linked by horizontal lines. ***P < 0.001, **P < 0.01, *P < 0.05.

Challenge experiments were conducted to determine the protective effects of SIRV in mice. Mice immunized with phosphate‐buffered saline (PBS) and the control vectors rSC0119(pS0018) or rSC0120(pS0018) died within 3 d of the challenge. All mice immunized with rSC0120(pS‐SaoA^ACA−^) survived the challenge with the heterologous or heterologous strains (**Figure** **7**A). However, immunization with rSC0119(pS‐SaoA^ACA−^) conferred 80%, 10%, 40%, and 20% protection against SS2, SS7, SS9, and SS1/2 challenges, respectively, in mice (Figure 7A). The SIRV strain rSC0120(pS‐SaoA^ACA−^) conferred significantly better protection against the heterologous strains SS2, SS7, and SS1/2 than rSC0119(pS‐SaoA^ACA−^). Histological analysis of lung tissue sections from PBS‐and rSC0119(pS‐SaoA^ACA−^)‐immunized mice demonstrated moderate hyperemia and inflammatory exudation, whereas tissue sections of rSC0120(pS‐SaoA^ACA−^)‐immunized mice displayed only minor histopathological lesions (Figure 7B), which were not significantly different from those of the unchallenged control. Brain tissue sections revealed typical features of meningitis, such as meningeal thickening, meningeal hemorrhaging, and neutrophilic infiltration in mice immunized with PBS and rSC0119(pS‐SaoA^ACA−^). However, such histopathological lesions were not observed in the rSC0120(pS‐SaoA^ACA−^)‐immunized group (Figure 7C).

The pig protection mode is often less effective than the mouse model when compared directly.^[^ ^25^ ^]^ Hence, we used vaccinations for further tests in a pig model. One 1.8 × 10^8^ – 7.6 × 10^8^ CFU for different *S. suis* serotype strain challenges, rSC0119(pS‐SaoA^ACA−^) provided only 16.7% protection against SS2 and no protection against challenges with SS7, SS9, and SS1/2 (Figure 7D). In contrast, rSC0120(pS‐SaoA^ACA−^) conferred 100% protection against SS2, SS7, SS1/2, and. There was an 83.3% protection against SS9 (Figure 7D). These results suggest that the SIRV strain rSC0120(pS‐SaoA^ACA−^) can provide efficient cross‐protection against multiple serotypes of *S. suis* in the target animal model. In addition, the brain and lung tissues of pigs immunized with rSC0120(pS‐SaoA^ACA−^) did not show significant lesions after challenge with multiple serotypes, whereas the lungs and brains of pigs immunized with rSC019(pS‐SaoA^ACA−^) showed varying degrees of hemorrhaging and inflammatory cell infiltration (Figure 7E,F). These results suggest that rSC0120(pS‐SaoA) not only protected pigs from mortality due to multiple serotype *S. suis* challenge but also protected pigs from pneumonia and meningitis caused by *S. suis*.

## 3 Discussion

Live bacterial vectors must allocate limited metabolic resources to synthesize sufficient antigens to induce the desired immune responses without compromising colonization.^[^ ^12^ ^]^ However, excessive colonization often leads to unwanted emissions, raising concerns regarding biocontainment.^[^ ^40^ ^]^ Therefore, programmed antigen production and lysis after vector colonization inside the host are considered optimal states for a live vector.^[^ ^4^, ^13^ ^]^ Based on the fact that 96% of *Salmonella* genes contain ACA triplets (Table S1, Supporting Information), we hypothesized that delayed MazF synthesis regulated by arabinose would enable the antigen to become the dominant protein in the cell, provided that the ACA triplet in the antigen gene was removed, ultimately leading to lysis following *Salmonella* colonization (Figure 1A). As expected, the SIRV strain showed similar colonization to the non‐SIRV strain during the early stages of colonization. However, colonization was reduced in the later stages owing to lysis while achieving higher antigen production and reduced background protein production (Figures 2J and 4A–D; Figure S4A–D, Supporting Information). Importantly, consistent with phenotypes previously reported for the single protein production (SPP) system,^[^ ^15^, ^41^ ^]^ antigen production persisted even after lysis of the SIRV strain (Figure 4A–D; Figure S4A–D, Supporting Information). These phenotypes confer the *Salmonella* vector with in vivo antigen delivery and self‐lysis, thereby balancing immunogenicity and safety.

The outer membrane of bacteria binds foreign antigens in the cytoplasm, resulting in low antigen recognition and presentation efficiency.^[^ ^3^, ^4^, ^10^ ^]^ Here, we observed that the SIRV system mediated the formation of a transmembrane channel in bacteria, resulting in a significant release of antigens that were originally bound within the bacteria (Figures 2E,F and 4A–H). In addition to the mechanisms of membrane damage mediated by MazF, which have already been reported,^[^ ^16^, ^17^, ^18^ ^]^ the downregulation of the cell wall synthesis‐related genes *murA*,^[^ ^42^ ^]^ *alr*, *dadX*,^[^ ^43^ ^]^ and *asd*,^[^ ^40^ ^]^ all of which contain ACA triplets, could potentially contribute to membrane breakage under the pressure of the SIRV system (Table S2, Supporting Information). Deleting membrane synthesis‐related genes and exploring the production of these proteins in future research may help further elucidate the causality between MazF and membrane pores. Although the mechanism by which the SIRV system mediates membrane breakage in *Salmonella* remains to be addressed, our results prove that the foreign antigen breaks through the constraints of the membrane structure and bridges the delivery of foreign cargo to the extracellular space.

Several studies have shown that *Salmonella* is involved in activating the cGAS‐STING axis, even though it is an intracellular bacterium.^[^ ^44^ ^]^ This may be due to the fact that *Salmonella* DNA is enclosed by at least two layers of membrane, bacterial membrane/wall and the cellular membrane structures it recruits.^[^ ^4^, ^13^, ^34^ ^]^ We observed that the membrane of rSC0120 was disrupted and the cytoplasmic contents were released concomitantly with MazF production (Figure 2E–G). These cell contents contained DNA that stimulates cGAS‐STING pathway, as evidenced by increased cGAS activity, up‐regulated cGAMP and p‐STING production, and increased IFN‐β secretion in both murine and porcine macrophages (Figure 3; Figure S3, Supporting Information). These data indicate that the SIRV system enhances the interaction between the bacterial vector and the cytoplasmic surveillance pathway, which is beneficial for fully mobilizing the immune system of the host.

Although the STING pathway has garnered tremendous interest in the fields of cancer treatment and vaccine adjuvants, most STING agonists have only been tested in mice and have not been implemented in other animals.^[^ ^45^ ^]^ In addition, some STING agonists are effective in mice but not in humans, limiting their clinical application.^[^ ^46^ ^]^ Strain rSC0120 can stimulate cGAS‐STING axis activation and IFN‐β production in both murine and porcine macrophages, mice, and pigs (Figure 3; Figure S3, Supporting Information), suggesting that activation of the STING pathway by the SIRV strain has a broad host range. The cytosolic surveillance of cGAS‐STING exists in most vertebrates.^[^ ^47^ ^]^ Therefore, we should consider the SIRV system as a STING agonist with multi‐animal species adaptability and broad application prospects.

Vaccines adjuvanted with STING agonists mediate robust immune defense against infections and cancer by promoting the maturation of antigen‐presenting cells (APCs) and activation of T cells.^[^ ^35^ ^]^ Our data further confirmed these conclusions; the co‐stimulatory molecules CD40 and CD86 and the levels of IL‐12p70 and IL‐6 induced by rSC0120 in BMDCs and BMDMs were significantly higher than those induced by rSC0119 (Figure 4I–K; Figure S5, Supporting Information). The versatile cytokines IL‐6 and IL‐12p70 produced by APCs are critical for regulating immune responses by promoting T and B cell proliferation, differentiation, survival, and antibody production.^[^ ^48^, ^49^ ^]^ Compared to rSC0119(pS‐SaoA^ACA−^), rSC0120 (pS‐SaoA^ACA−^) induced significant upregulation of SaoA‐specific humoral, cellular, and mucosal immune responses in both mice and pigs (Figure 6). These data demonstrate that SIRV is an effective vector for inducing APCs maturation, proliferation, and the differentiation of antigen‐specific T cells, which can be translated into an improved adaptive immune response.

As an important PAMP, the interactions of flagellin with the host seem to have contradictory consequences. On the one hand, they can stimulate and activate the immune system by stimulating TLR5 and the inflammasome.^[^ ^37^, ^38^ ^]^ On the other hand, they also cause excessive inflammation and even pyroptosis of immune cells, leading to the suppression of immune responses.^[^ ^50^ ^]^ The synthesis of flagella in strain rSC0120 gradually decreased as the SIRV system operated, but not in rSC0119 (Figure 5A–C). Accompanied by these results, rSC0120 induced less self‐cleavage of the inflammasome and secretion of proinflammatory cytokines IL‐1β and IL‐18 than rSC0119 did (Figure 5D,E). These results demonstrate that SIRV could induce appropriate activation of the inflammasome in APCs, but not overactivation. Importantly, activation of the STING pathway results in less inflammation than activation of the TLR or inflammasome pathways.^[^ ^51^ ^]^ Moderate but not excessive activation of the inflammasome promotes the expression of costimulatory molecules on APCs, thus improving the immune response.^[^ ^52^ ^]^ Based on our data, it can be concluded that SIRV‐mediated modest activation of the inflammasome and activation of the cGAS‐STING‐IFN‐β axis synergistically promote APCs maturation. Therefore, SIRV enables *Salmonella* to be a safe and effective delivery platform by enhancing the properties of STING agonists and circumventing the side effects caused by flagella in *Salmonella*.

STING agonists have shown tremendous potential for the prevention or treatment of viral infections^[^ ^51^, ^53^ ^]^; however, few studies have used them in the development of vaccines targeting extracellular pathogens. The Th‐17 response has been shown to play a critical role in the clearance of extracellular pathogens such as *Streptococcus pneumoniae* ^[^ ^54^ ^]^ and *Mycoplasma*.^[^ ^55^ ^]^ Compared to rSC0119(pS‐SaoA^ACA−^), rSC0120(pS‐SaoA^ACA−^) mediated a significant upregulation of SaoA‐induced IL‐17A in both mice and pigs (Figure 6G,H). These data are consistent with previous reports showing that activation of the STING pathway can promote a Th17‐biased immune response.^[^ ^56^ ^]^ Therefore, our data highlight the importance of activating the STING pathway to combat extracellular pathogens and demonstrate that SIRV is a highly promising in vivo delivery platform for targeting intracellular or extracellular pathogens.

Although OPA is believed to be directly related to protection, few studies have reported cross‐reactive OPA antibodies in the context of the urgent need to develop a universal vaccine against *S. suis*.^[^ ^25^ ^]^ STING agonists have been shown to enhance antibody‐dependent CTL responses to influenza vaccines, highlighting the advantages of the STING pathway in mediating FcR functions.^[^ ^53^ ^]^ Relative to rSC0119(pS‐SaoA^ACA−^), rSC0120(pS‐SaoA^ACA−^) induced OPA antibodies against homologous SS2 as well as heterologous serotypes SS7, SS9, and SS1/2 (Figure 6D,E). These results could be attributed to the strong adjuvant activity of rSC0120, which mediates the broad activation of more conserved OPA antibody epitopes involved in the interaction between the antibody and FcR.

Recently, meningitis caused by *S. suis* in humans has become a major public health concern in several Asian countries.^[^ ^22^, ^23^, ^24^ ^]^ However, few studies have reported vaccines that prevent *S. suis*‐induced meningitis. Pathological examination revealed no histopathological damage to the brain tissue of mice or pigs immunized with rSC0120(pS‐SaoA^ACA−^) (Figure 7C,F). These data indicated that SIRV can effectively prevent brain damage caused by *S. suis*. Considering the similarities in the immune systems and organs between humans and pigs,^[^ ^57^ ^]^ SIRV has the potential to prevent meningitis or other brain damage caused by *S. suis* in humans.

It is worth noting that our platform is a dynamic production process, allowing for evolution over time. Consequently, any issues related to production efficiency or preventive/therapeutic performance of the designed protein cargos can be addressed through targeted evolution‐based improvement strategies.

## 4 Experimental Section

### Animals and Ethics Statement

The Jiangsu Laboratory Animal Welfare and Ethics Standards of the Jiangsu Administrative Committee of the Laboratory approved all experimental methods. The Department of Science and Technology of Jiangsu Province granted the license numbers SYXK (SU) 2021‐0026 and SCXK (SU) 2017‐0007 for animal supervision. Six‐week‐old female BALB/c mice were purchased from Yangzhou University Comparative Medicine Center. Three‐week‐old female Landrace/White mixed‐breed pigs were purchased from Jiangsu Lihua Animal Husbandry Co., LTD, Jiangsu Province, China. Both mice and pigs were seronegative against SaoA or *S*. Choleraesuis OMPs, as evidenced by serum IgG titers that were less than the cutoff value detected using enzyme‐linked immunosorbent assay (ELISA).

### Bacterial Strains, Plasmids, and Culture Conditions

The bacterial strains and plasmids used in this study are listed in Table S4 (Supporting Information). Plasmid pYA3493 was an Asd^+^ vector. Plasmid pS0018 is a derivative of pYA3493 in which the ACA sequences in *asd* were removed without alternating the amino acid sequence. SaoA is from SS2. The amino acid homologies between SS2 and SS7, SS2 and SS9, and SS2 and SS1/2 were 94.2%, 98.5%, and 100%, respectively. The plasmid pS‐SaoA^ACA+^ derived from pS0018, carries an SS2 wild‐type *saoA* gene containing 16 ACA triplets. The plasmid pS‐SaoA^ACA−^, derived from pS0018, carries the SS2 *saoA* gene without the ACA triplets. All oligonucleotides and/or gene fragments were commercially synthesized. Primers used are listed in Table S5 (Supporting Information). With the donor strain χ7213, as previously reported,^[^ ^58^ ^]^ suicide vectors were used to insert mutations into *S*. Choleraesuis strains by conjugational transfer for the creation of unmarked deletion and deletion‐insertion mutations.^[^ ^59^ ^]^ All *S. suis* strains were grown in Todd–Hewitt broth (THB; BD Biosciences). *E. coli* and *S*. Choleraesuis strains were grown in Luria‐Bertani (LB; Oxoid) broth, Nutrient Broth (NB; BD Difco) or plated onto LB agar at 37 °C. When necessary, chloramphenicol (25 mg mL^−1^), kanamycin (50 mg mL^−1^), ampicillin (100 mg mL^−1^), 2,6‐diaminopimelic acid (DAP; 50 mg mL^−1^), arabinose (0.2% w/v), and D‐lactose (1% w/v) were used.

### Mammalian Cell Culture

Cells were maintained in a humidified incubator at 37 °C, 5% CO_2_. RAW264.7, 3D4/21, and their corresponding cGAS KO macrophages were grown in Dulbecco's modified Eagle's medium (DMEM; GIBCO) supplemented with 10% fetal bovine serum (FBS; GIBCO) and 100 U penicillin‐streptomycin. Bone marrow‐derived dendritic cells (BMDCs) or BMDMs were differentiated from bone marrow isolated from BALB/c mice using a previously described protocol.^[^ ^53^ ^]^ In brief, BMDCs were grown in Roswell Park Memorial Institute (RPMI) 1640 medium (GIBCO) supplemented with 10% (v/v) FBS, 100 U penicillin‐streptomycin, IL‐4 (500 U/mL, Abcam), and GM‐CSF (1, 000U/mL, R&D Systems). BMDMs were grown in RPMI 1640 medium (GIBCO) supplemented with 10% (v/v) fetal bovine serum (FBS), 100 U penicillin‐streptomycin, and M‐CSF (10 ng/mL, R&D Systems). The culture medium was refreshed every 2 d. Cells were differentiated for 5–8 d in 15 cm Petri dishes. Cell purity was at least 85% CD11c^+^ (BMDCs) or 85% F4/80^+^ (BMDMs), and the cells were seeded into tissue culture dishes the day before infection.

### Transcriptional Analysis of Mutation‐Adjacent Genes

*S*. Choleraesuis strains were grown in NB medium with 0.2% (w/v) arabinose and 0.2% (w/v) mannose for the logarithmic growth period. Total RNA was extracted from each strain. One microgram of total RNA was reverse‐transcribed into cDNA, which served as a template to analyze the mRNA expression levels of *sh2895*, *ygcA*, *yggL*, *sprT*, *nirC*, and *yhfL* using the primers listed in Table S5 (Supporting Information). Relative gene expression data were calculated against mouse GroEL expression, and the analyses were carried out using the 2^^‐ΔΔCt^ method.

### Generation of Polyclonal Antibodies against MazE and MazF

Recombinant His6‐MazE or His6‐MazF was synthesized in BL21(DE3) cells harboring the plasmids pET28a‐MazE or pET28a‐MazF. Recombinant proteins were purified using His. Bind^@^ purification kit (Novagen) according to the manufacturer's instructions. Polyclonal antibodies were raised in rabbits using the relevant recombinant proteins.

### Characterization of Regulatory Elements in SIRV

Overnight cultures of strains rSC0120 and rSC0119 carrying plasmid pS‐SaoA^ACA−^ were grown in NB medium with 0.2% (w/v) arabinose for 12 h. The cultures were washed thrice with NB medium without arabinose and then diluted 1:100 in NB medium without arabinose. This process was repeated every 12 h for 14 passages. Samples were taken at each dilution and normalized to an optical density at 600 nm (OD_600_) of 0.6 for sodium dodecyl sulfate‐polyacrylamide gel electrophoresis (SDS‐PAGE) followed by InstantBlue® (Abcam) staining or western blot analysis using anti‐MazE, anti‐MazF, anti‐LacI (Abcam), anti‐SaoA^[^ ^27^ ^]^ or anti‐GroEL (BD Biosciences) antibodies in accordance with the previous procedure. Imprinting was performed using a ChemiDoc XRS + (Bio‐Rad). SDS‐PAGE and western blotting are representative of at least three independent experiments. Relative densitometry bands were analyzed using the ImageJ software from the National Institute of Health (NIH) and normalized according to the densitometry of GroEL.

### TEM and SEM

Bacterial samples were fixed with 2.5% (v/v) glutaraldehyde for 12 h at 4 °C. Subsequently, the samples were washed thrice with PBS for 15 min each and fixed with 2% (v/v) osmic acid (Sigma) for 2 h, followed by washing thrice with PBS for 10 min each time. Each dehydration phase lasted 15 min in 50%, 70%, 80%, 90%, and 100% (v/v) ethanol. Before soaking overnight in the 100% SPI‐Pon 812 resin, the samples were soaked in 1:1, 1:2, or 1:3 ratio mixes of SPI‐Pon 812 resin (SPI) and acetone. Before microscopy, samples were sectioned and stained with uranyl acetate (Sigma), and lead citrate (Sigma) after being polymerized at 65 °C for 50 h. Samples preparation for SEM (GeminiSEM 300, Carl Zeiss) was accomplished by centrifuging an induced bacterial solution at 1500 RPM for 10 min, followed by fixation with 2.5% (v/v) glutaraldehyde for 2 h at 4 °C. Three 10‐min washes were performed with PBS. 10‐min dehydration stages were conducted in 50%, 70%, 80%, 90%, and 100% (v/v) ethanol, a 1:1 combination of 100% ethanol and tert‐butanol (Sigma‐Aldrich); and pure tert‐butanol (Sigma‐Aldrich). After ≈4 h of lyophilization, the samples were placed on a microscope substrate, coated with gold particles, and analyzed.

Minor alterations were made to previously reported procedures while preparing samples for fluorescence microscopy.^[^ ^60^ ^]^ In brief, 20 µL of concentrated cells were added to 4 µL of a stain mixture comprising DAPI (2.5 µg mL^−1^; Invitrogen), SYTOX Green (5 µm; Invitrogen), and FM4–64 (20 µg mL^−1^; Invitrogen) prepared in 1 × PBS. The stained samples were fixed on glass slides and photographed using a Leica SP8 Laser Scanning Confocal Microscope at 1000 × magnification. The medial focal planes were deconvolved using LAS X software (Leica).

### Plasmid Stability of *S*. Choleraesuis Strains

Plasmid stability was assessed as previously described.^[^ ^61^ ^]^ Briefly, 3 µL overnight culture of *Salmonella* strains carrying vector pS0018 or pS‐SaoA^ACA−^ in LB broth were inoculated into 3 mL of LB medium with 0.2% arabinose and 50 µg mL^−1^ DAP as nonselective conditions for 12 h with rotation at 37 °C. The process was repeated every 12‐h period for five passages, with each passage consisting of 10 generations, for a total of 50 generations. At each passage, a sample of the culture was serially diluted and plated onto LB‐arabinose‐DAP plates. Subsequently, randomly selected 100 single colonies were patched on LB agar plates without any supplements (selective media) and on LB agar plates containing either arabinose or DAP (differential selective media). Plasmid stability was determined as the percentage of colonies (out of 100 selected colonies) grown on selective media after every passage. After five passages, randomly selected colonies were evaluated for the presence of the *saoA* gene and the ability of the isolate to produce SaoA.

### Virulence

The strains C78‐3, rSC0119(pS‐SaoA^ACA−^), and rSC0120(pS‐SaoA^ACA−^) were grown in LB medium with 0.2% (w/v) arabinose and 0.2% (w/v) mannose for a logarithmic growth period. Bacteria were obtained by centrifuging at 8000 RPM for 10 min at 25 °C and then resuspended in PBS for i.p. or oral routes, or density measurement. Within each treatment group, each subset of ten 6‐week‐old BALB/c mice was challenged with one of the 10‐fold dilutions of bacteria from 10^1^ to 10^7^ CFU. The control group consisted of mice treated with PBS. The mice were followed‐up for 3 weeks, and their deaths were documented to calculate the LD_50_ of the strains using the Reed and Muench method.^[^ ^62^ ^]^

### HPLC Analysis

*Salmonella* strains were passaged as described above. Arabinose was added only during the first passage and was subsequently excluded from the subculture medium. Each culture was diluted and plated onto LB medium with 0.2% (w/v) arabinose to measure the bacterial density. The culture was centrifuged at 12 000 rpm min^−1^. The supernatant was collected and filtered through a 0.45 µm filter. Bacterial cells were collected and resuspended in an 80% acetonitrile (Sigma)‐water solution and normalized to an OD_600_ value of 0.5 to ensure uniform bacterial density in the suspension per unit volume. The suspension was then subjected to ultrasonication subsequently. A standard curve was plotted based on the relationship between the peak area and concentration of the arabinose standard. Subsequently, a linear equation was derived. An LCQ Deca XP MAX chromatograph (Thermo Fisher Scientific) was used for detection. The chromatographic column used was an amino column (Thermo Fisher Scientific) with dimensions of 4.6 mm × 250 mm and a particle size of 5 µm. The mobile phase consisted of an 80% acetonitrile‐water solution. The column temperature was maintained at 30 °C, and the injection volume was 10 µL. The flow rate was set at 0.8 mL min^−1^.

### Colonization of Mice with SIRV

Six‐week‐old female BALB/c mice were fasted from food and water for 4 h prior to inoculation with oral *S*. Choleraesuis strains. The strains were subcultured to an OD_600_ of 0.85 from a stationary culture in LB media supplemented with 0.2% (w/v) arabinose and 0.2% (w/v) mannose for 16 h at 37 °C. Strains rSC0119 with either pS‐SaoA^ACA−^ or pS0018 and rSC0120 with either pS‐SaoA^ACA−^ or pS0018 were orally administered to the mice with 20 µL containing 1 ± 0.2 × 10^9^ CFU suspended in PBS. Food and water were returned to the mice 30 min later. Aseptically harvested Peyer's patches, spleens, and livers were collected at indicated time points. To assess colonization and persistence, tissues were homogenized and plated on Luria‐Bertani (LB) agar with 0.2% (w/v) arabinose.

### Subcellular Localization of Chromosomal and Plasmid DNA in *S*. Choleraesuis Strains

Total DNA from the relevant samples was extracted using a DNAzol reagent kit (Invitrogen) following the manufacturer's instructions. Real‐time quantitative (q)RT‐PCR was performed to determine the copies of the *S*. Choleraesuis *stn* gene and the pS‐SaoA^ACA−^ plasmid. qRT‐PCR was conducted using the ChamQ Universal SYBR qPCR Master Mix (Thermo Fisher Scientific); the primers are listed in Table S5 (Supporting Information). Each group was comprised of three replicates. Using a 7500 fast real‐time PCR apparatus (ABI, US), standard measurements of the pMD19T‐*stn* and pS‐SaoA^ACA−^ plasmids in the range of 0–10^9^ copies were performed. The mean Ct values were used to calculate the copies of the *stn* gene and pS‐SaoA^ACA−^ plasmid, which were then divided by the bacterial density, periplasm volume, and supernatant volume and converted to viral copies per 10^9^ CFU of bacteria per milliliter of periplasm or per milliliter of supernatant.

### α‐Gal and β‐Gal Assays

Overnight cultures of *S*. Choleraesuis strains were grown in NB medium with 0.2% (w/v) arabinose for 12 h. The cultures were washed thrice with NB medium without arabinose and then diluted 1:100 in NB medium without arabinose. This process was repeated every 12 h for ten passages. Serial dilutions of the test samples were plated onto LB agar, and then cultivated for 1 d, additionally, at 37 °C. The cytoplasmic, periplasmic, and supernatant samples were analyzed with α‐gal (Solarbio) assay kit or β‐gal assay kit (Solarbio) in normalized bacterial samples of equal density or volume.

### Transcription of Peptidoglycan Synthesis Genes in SIRV

The strains rSC0119(pS0018), rSC0120(pS0018), rSC0119(pS‐SaoA^ACA−^), and rSC0120(pS‐SaoA^ACA−^) were cultured continuously in NB medium with decreasing arabinose, according to the method described above. Total RNA was extracted from the 1 st, 5 th, and 10 th passages. One microgram of total RNA was reverse transcribed into cDNA, and the cDNA was used as a template to analyze the mRNA levels of *murA*, *alr*, *dadX*, and *asd* using the primers listed in Table S5 (Supporting Information). Relative gene expression data were calculated against mouse GroEL expression, and the analyses were carried out using the 2^^‐ΔΔCt^ method.

### BrdU Assay

To label *Salmonella* DNA, bacteria were grown in 0.15% (w/v) BrdU (Sigma) for 3 d in a dark room. BrdU‐labeled cells were used to infect RAW264.7, at a multiplicity of infection (MOI) of 10 for 24 h. Cells were washed once with PBS and then lysed in PBS containing 0.02% (w/v) digitonin for 15 min on ice at 1, 6, 12, and 24 h post‐infection. Supernatants were applied on Protein‐G dyna beads (Roche) that were conjugated with anti‐BrdU antibodies after filtering through a 0.45‐micron filter (Sigma). The beads were rinsed twice with PBS with Tween (PBST) after an overnight incubation. The bound BrdU‐labeled DNA was liberated by boiling the beads for 10 min. Isopropyl alcohol was used to precipitate DNA, and the purified DNA was used as a template to detect the *S*. Choleraesuis *stn* gene. Relative gene expression data were calculated against mouse GAPDH expression, and the analyses were performed using the 2^^‐ΔΔCt^ method.

### CRISPR‐Cas9 Knockout

The CRISPR‐Cas9 system was used for genetic manipulation. Target sequence‐specific double‐stranded oligonucleotides were inserted into the lenti‐CRISPR‐V2 vector and co‐transfected into HEK293 cells, along with packaging plasmids. The viruses were isolated 2 d after transfection and used to infect RAW264.7 cells. Cells were screened with puromycin (1 µg mL^−1^) for 6 d, 24 h after transfection. Subsequently, in 96‐well plates, cells were serially diluted twice to generate oligoclonal cell lines for gene deletion. Protein knockdown was further functionally confirmed by evaluating phenotypic responses to pertinent stimuli, as described below. Serial dilutions were used to produce single clonal knockout cells, and Sanger sequencing was used to confirm these results. The sequences targeting mouse cGAS are listed in Table S4 (Supporting Information).

### Macrophage Infection

RAW264.7, 3D4/21, and their corresponding cGAS KO cells were infected as previously described, with a few modifications.^[^ ^63^ ^]^ Briefly, *S*. Choleraesuis cultures were rinsed thrice with Phosphate‐Buffered Saline (PBS) and resuspended in DMEM supplemented with 10% (v/v) FBS. The monolayers were coated with the bacterial suspension and centrifuged for 10 min at 1000 RPM. Infected plates were spun at 1000 RPM for 5 min to synchronize the host cell adhesion. Subsequently, the cells were infected at 37 °C and 5% CO_2_. After removing the extracellular bacteria with gentamycin, the cells were washed twice with PBS and maintained in DMEM. Interferon‐stimulatory DNA (ISD) was transfected into cells as a positive control using lipofectamine 2000 (Thermo Fisher Scientific) at a concentration of 2 µg mL^−1^. ISD was prepared from equimolar amounts of sense and antisense DNA oligonucleotides. The sense strand sequences are listed in Table S5 (Supporting Information). The oligonucleotides were heated at 95 °C for 5 min and cooled to room temperature. Supernatants were collected for cytokine measurements (IFN‐β) at 12 h after infection. A Micro bicinchoninic acid (BCA) protein kit (Beyotime) was used to measure protein concentration, and equal amounts of protein were used for western blot analysis. Samples were detected with anti‐cGAS (Proteintech), anti‐STING (Cell Signaling Technology), and anti‐phospho‐STING (Cell Signaling Technology) antibodies, and normalized to GAPDH using an anti‐GAPDH antibody (Abcam). Phospho‐STING (Ser365) antibodies for RAW264.7 cells and Phospho‐STING (Ser366) antibodies for 3D4/21 cells. An anti‐rabbit IgG‐horseradish peroxidase (HRP) coupled antibody (Cell Signaling Technology) was used as the secondary antibody.

### Liquid Chromatography‐Mass Spectrometry (LC‐MS) of cGAMP Synthesis Assay

RAW264.7 cells were infected by rSC0119(pS0018), rSC0120(pS0018), rSC0119(pS‐SaoA^ACA−^), and rSC0120(pS‐SaoA^ACA−^) according to the above, respectively. At 12 h after infection, the medium was aspirated from the cells, and digitonin (5 µg mL^−1^) was added to the cells for permeabilization in a buffer solution (30 mm 4‐(2‐hydroxyethyl)−1‐piperazineethanesulfonic acid [HEPES], 50 mm KCl, 2 mm MgCl_2_, 0.1 mm dithiothreitol [DTT[, 60 mm sucrose, and 0.5% bovine serum albumin [BSA], pH 7.0). The cell samples were diluted with 50% (v/v) acetonitrile and centrifuged at 10 000 RPM for 20 min. Filtering the supernatant via a 0.22 µm ultrafiltration filter (Millipore). Vacuum drying was followed by reconstituted cGAMP being ultrasonically processed for 30 min at 25 °C in 0.5 mL of 50% (v/v) acetonitrile. The supernatant was collected for LC‐tandem mass spectrometry (MS/MS) analysis after centrifugation at 10 000 RPM. LC‐MS/MS analysis was conducted on a Acquity UPLC I‐class/VION IMS QTOF mass spectrometer (Waters). An EC 150/2.0 NUCLEODUR C18 pyramid chromatography column (3 µm) was used for GAMP separation with a flow rate of 0.4 mL min^−1^ and column temperature of 30 ˚C. The injection volume was set to 5 µL. The mobile phase consisted of a 0.2% (v/v) aqueous solution of formic acid and acetonitrile. Under mobile phase conditions, each sample was analyzed for 8 min. The mass parameters were as described below: ion spray voltage was 5000 V, ion source temperature was 500 ˚C, the declustering voltage was 90 V, the intake voltage was 7 V, ion source gas 1 was 65 psi, ion source gas 2 was 65 psi, curtain gas was 35 psi. The quantitative ion pairs were 675/524 (parent ion/daughter ion).

### BMDCs and BMDMs Analysis

BMDCs and BMDMs were cultured with different strains for 48 h at an MOI of 10. Supernatants were collected to analyze the levels of IL‐6 and IL‐12p70. Cells were stained with CD11c‐FITC (BD Biosciences), F4/80‐PE (BD Biosciences), CD86‐PE (BD Biosciences), and CD40‐APC (BD Biosciences) and analyzed using a FACSAria SORP cytometer (BD Biosciences).

### Antigen Release from Salmonella

The relevant bacterial strains were administered to BMDCs in 6‐well plates at an MOI of 10. After removing the extracellular bacteria by gentamycin treatment and washing, BMDCs were cultured for 24 h. One set of dishes was fixed, permeabilized, and stained with anti‐SaoA^[^ ^27^ ^]^ and anti‐CD11c‐FITC (BD Biosciences) antibodies at 6, 12, and 24 h post infection. A PE Mouse anti‐rabbit IgG Detector (BD Biosciences) was used as the secondary antibody and analyzed using a FACSAria SORP cytometer (BD Biosciences).

### Phenotypic Analysis of Flagella in SIRV

*S*. Choleraesuis strains were passaged in NB medium, and arabinose (0.2% w/v) and mannose (0.2% w/v) were added to the primary culture according to the method described above. The flagella of each passage of the strains were analyzed. Flagella were analyzed by transmission electron microscopy (TEM), and the number of flagella per cell was counted manually for ten cells per strain at each time point. To check the motility, 2 µL of each strain with OD_600_ normalized to 0.1 was spotted on LB media with 0.3% (w/v) agar. After 6 h of motionless incubation at 37 °C, plates were scanned using an image scanner (Canon).^[^ ^63^ ^]^ The motility zone was the average of three independent measurements using ImageJ software. Data are representative of three independent biological replicates. Bacterial cells were collected, separated by SDS‐PAGE, and analyzed using western blotting. The blots were probed with mouse anti‐GroEL (BD Biosciences) or mouse anti‐FliC antibodies (BioLegend). Imprinting was performed using a ChemiDoc XRS + (Bio‐Rad). SDS‐PAGE and western blotting are representative of at least three independent experiments. Relative densitometry was performed as described previously.

### Inflammasome Assay

*S*. Choleraesuis strains were grown to the logarithmic phase, collected, and resuspended in cell culture medium for DC infections at a MOI of 10. BMDCs were treated with 100 mM QVD‐oPh (Abcam) for Caspase‐1 inhibition 1 h before infection. The cell supernatants were collected at 12 h post‐infected to analyze the levels of IL‐1β and IL‐18. The cell supernatants were treated with ice‐cold 10% TCA for albumen precipitation at 1, 6, 12, and 24 h post‐infection to analyze secreted Caspase‐1 by western blotting. Anti‐Caspase‐1 antibody (Abcam) was used as the primary antibody. An anti‐rabbit IgG‐horseradish peroxidase (HRP) coupling antibody (Cell Signaling Technology) was used as a secondary antibody and normalized to GAPDH (Abcam).

### Cytokine Quantification

Cytokine levels in cell culture supernatants, mouse or pig were analyzed using commercial ELISA kits from Abcam (Porcine IFN‐β, Mouse IL‐18, and Mouse IFN‐β Kit), or from BD Biosciences (Mouse IL‐6, Mouse IL‐12p70, and Mouse IL‐1β) according to the manufacturer protocols.

### Mouse and Pig Immunization and Challenges

Mice were randomly divided into five groups (50 mice per group) and orally inoculated. With 1 ± 0.2 × 10^9^CFU of rSC0119(pS0018), rSC0120(pS0018), rSC0119(pS‐SaoA^ACA−^), rSC0120(pS‐SaoA^ACA−^), or 20 µL PBS, respectively. Pigs were randomly divided into five groups (30 pigs per group) and inoculated through oral route with 1 ± 0.2 × 10^10^ CFU of rSC0119(pS0018), rSC0120(pS0018), rSC0119(pS‐SaoA^ACA−^), rSC0120(pS‐SaoA^ACA−^) or 5 mL PBS blank control, respectively. Mice and pigs were boosted with the same dose 3 weeks later. Sera were collected at 24 h, 3 and 5 weeks following first inoculation, and vaginal wash (from mice) and nasal wash (from pig) samples were obtained and stored at −20 °C. The production of IFN‐β in serum collected 24 h after the first vaccination was analyzed. Vaginal or nasal wash IgA responses to SaoA and serum IgG against SaoA were assayed using ELISA, as described above. Five weeks after the first inoculation, ten mice randomly selected from each group were i.p. challenged with 1.5 × 10^8^ CFU of SS2 (12.5× the LD_50_), 1.2 × 10^8^ CFU (12× the LD_50_) of SS7, 2.7 × 10^8^ CFU (11.7× the LD_50_) of SS9 or 5.1 × 10^8^ CFU (11.3× the LD_50_) of SS1/2. Five weeks after the first inoculation, six pigs randomly selected from each group were i.v. challenged with lethal doses of SS2, SS7, SS9, or SS1/2 (SS2: 2.3 × 10^8^ CFU; SS7: 7.6 × 10^8^ CFU; SS9: 5.6 × 10^8^ CFU; SS1/2: 1.8 × 10^9^ CFU). The data from two independent replicates of the protection tests were similar and aggregated for analysis. Animals were observed twice daily for the appearance of clinical symptoms and mortality during the 14 d after the challenge. Mice with meningitis‐like symptoms, including anorexia, lethargy, and indifference, were euthanized. Pigs with clinical symptoms including dorsal head inclination, ataxia, nystagmus, convulsions lateral, and a body temperature exceeding 42 °C for 2 d, consecutively, were compassionately euthanized. A postmortem investigation was performed on all mice and pigs. When required, the brains and lungs of animals were fixed in 10% (v/v) formalin. Following paraffin wax embedding, slices of tissue (4 m) were stained with hematoxylin and eosin (H&E) using an H&E staining kit (Sangon Biotech) according to the manufacturer's guidelines and observed by optical microscopy to assess pathological alterations. The following pathological scores were assigned to the histological sections of each mouse or pig: For the lungs, the ranges were 0 for normal, 1 for congestion, 2 for interstitial thickening, 3 for inflammatory cell infiltration into the bronchial submucosa, and 4 for an abundance of inflammatory cell infiltration. For the brain, 0 for normal, 1 for congested, 2 for a few inflammatory cells infiltrating the meninges, 3 for a mass of inflammatory cells, and 4 for meningeal bleeding with significant inflammatory cell infiltration.

### ELISPOT Assays

ELISPOT plates were coated with anti‐mouse IFN‐γ, IL‐4 (BD Biosciences), or IL‐17A (Invitrogen) antibodies on the grounds of the manufacturer's protocols. The plates were activated with 10% (v/v) ethanol (Gibco) and incubated for 30 min in 10% (v/v) FBS (Gibco). For the detection of IFN‐γ, IL‐4, or IL‐17‐secreting CD4^+^ T cells from immunized mice. CD4^+^ T cells were prepared as previously described^[^ ^64^ ^]^ by negative selection using a CD4^+^ T cell Isolation Kit (Miltenyi Biotec) according to the manufacturer's protocol. CD4^+^ T cells were seeded at 1 × 10^6^ per well at 37 °C in 5% CO_2_ for 48 h with SaoA protein. The cells were removed and incubated with biotinylated anti‐mouse IFN‐γ (BD Biosciences), IL‐4 (BioLegend), or IL‐17A (Abcam)‐specific antibodies. The plates were washed thrice with PBS before administration of the streptavidin‐HRP conjugate and developed with a ready‐to‐use AEC substrate. After drying, an Immune Spot Reader (Cellular Technology Ltd.) was used to determine the number of spots produced. Data were obtained from triplicate wells.

### Acquired Immune Cytokines in Pigs

Seven days after the booster was administered through oral immunization, porcine splenocytes were isolated from immunized pigs and grown in RPMI‐1640 supplemented with 10% FBS (GIBCO) and IL‐2 (Abcam; 100U/mL). The cells were seeded at 1 × 10^6^ cells per well with SaoA protein in a humidified incubator at 37 °C and 5% CO_2_ for 48 h. Cells were then harvested for Total RNA extracted from the cells and used extraction to determine the transcript levels of IL‐4, IFN‐γ, or IL‐17A with the primers listed in Table S4 (Supporting Information). Relative gene expression data were calculated against pig β‐actin expression, and the analyses were performed using the 2^^‐ΔΔCt^ method.

### qRT‐PCR

Following the manufacturer's protocols, total RNA was extracted using TRIzol® (Thermo Fisher Scientific). 1 µg total RNA was reverse transcribed into cDNA for qRT‐PCR. qRT‐PCR was performed using the Fast SYBR Green Master Mix (Thermo Fisher Scientific) on a 7500 Fast Real‐Time PCR Instrument (ABI, USA). Three biological replicates of qRT‐PCR were performed. GroEL, an internal reference for *S*. Choleraesuis; mouse GAPDH, an internal reference for RAW264. Seven cells, pig β‐actin, and an internal reference for pig tissues were used to standardize the expression values. The 2^^‐ΔΔCt^ method was used to evaluate the relative expression levels of the mRNAs of target genes.

ELISA was performed as previously described.^[^ ^27^ ^]^ Briefly, polystyrene 96‐well flat‐bottom microtiter plates (Corning) were coated with 100 ng per well of purified SaoA. Individual wells received a 100 µL volume of series diluted sample in triplicate and were then incubated for 2 h at 37 °C. Plates were incubated with goat anti‐mouse IgG (Sigma), goat anti‐mouse IgG1 (Abcam), goat anti‐mouse IgG2a (Abcam) or goat anti‐mouse IgA‐HRP coupling antibody (Abcam) when analyzing samples from mice, and incubated with goat anti‐pig IgG‐HRP (Sigma), mouse anti‐pig IgG1‐HRP, mouse anti‐pig IgG2‐HRP (Bio‐Rad) or goat anti‐pig IgA‐HRP coupling antibody (Abcam) when analyzing samples from pigs. The plates were developed using a 1‐Step^TM^ Ultra TMB‐ELISA kit (Thermo Fisher Scientific) and 5% H_2_SO_4_. An automatic ELISA plate reader (Model EL311SX; Biotek) was used to measure absorbance at 450 nm. Absorbance values 2.1‐fold greater than the baseline values of naive serum were regarded as positive.

### Opsonophagocytic Assay

The opsonophagocytic assay was performed as previously described.^[^ ^65^ ^]^ Briefly, serum from mice or pigs was collected for an opsonic killing assay on day 14 after boost immunization. Porcine polymorphonuclear leukocytes (PMNs) were isolated with Histopaque−1077 (Sigma‐Aldrich). Trypan blue staining was used to determine whether the cell mortality was less than 10%. 50 µL of the diluted sera samples were opsonized on log‐phase SS2, SS7, SS9, and SS1/2 for 15 min each at 37°C. In an equivalent volume (100 µL) of opsonized bacteria at a density of 1 × 10^7^ CFU mL^−1^, PMNs at a density of 1 × 10^7^ cells mL^−1^ were blended. Serial dilutions of the test samples were plated onto THB agar and then cultivated for an additional day at 37 °C after being incubated with PMNs for 1 h at that temperature. Bacteria were counted. The OPA titer was determined as the serum dilution that caused 50% of the test bacteria to die. Survival rate was calculated as the average of three individual CFU counts for each sample.

### Statistical Analyses

The statistical details of the specific experiment, including the statistical test used, number of samples, mean values, SEM, and p‐values, are described in the Figure legends. A student's *t*‐test was used for comparisons among groups. For comparison between each group with the mean of every other group within a dataset containing more than two groups, one‐way ANOVA with Tukey's multiple comparison test was used. Survival after challenge was compared using the log‐rank (Mantel‐Cox) test. ***P < 0.001, **P < 0.01, *P < 0.05, ^###^ *P* < 0.001, ^##^ *P* < 0.01, ^#^ *P* < 0.05, ns = not significant. Statistical analyses were conducted utilizing GraphPad Prism 8.0.

## Conflict of Interest

The authors declare no conflict of interest.

## Author Contributions

Y.‐a.L., R.C.III, and H.S. designed the research. Y.‐a.L., Y.S., and Y.Z. performed the research; H.S. contributed reagents and analytical tools. Y.‐a.L., Q.L., S.W., R.C.III, and H.S. analyzed the data. Y.‐a.L., S.W., R.C.III, and H.S. wrote the manuscript.

## Supporting information

Supporting Information

## Data Availability

The data that support the findings of this study are available from the corresponding author upon reasonable request.

## References

[1] H. Rohira , A. Arora , P. Kaur , A. Chugh , Appl. Microbiol. Biotechnol. 2023, 107, 3153. 37052636 10.1007/s00253-023-12512-5 PMC10099029

[2] J. L.‐S. Au , B. Z. Yeung , M. G. Wientjes , Z. Lu , M. G. Wientjes , Adv Drug Deliv Rev 2016, 97, 280. 26686425 10.1016/j.addr.2015.12.002 PMC4829347

[3] S. Dissanayake , W. A. Denny , S. Gamage , V. Sarojini , J Control Release 2017, 250, 62. 28167286 10.1016/j.jconrel.2017.02.006

[4] V. Raman , N. Van Dessel , C. L. Hall , V. E. Wetherby , S. A. Whitney , E. L. Kolewe , S. M. K. Bloom , A. Sharma , J. A. Hardy , M. Bollen , A. Van Eynde , N. S. Forbes , Nat. Commun. 2021, 12, 6116. 34675204 10.1038/s41467-021-26367-9 PMC8531320

[5] S. Behzadi , V. Serpooshan , W. Tao , M. A. Hamaly , M. Y. Alkawareek , E. C. Dreaden , D. Brown , A. M. Alkilany , O. C. Farokhzad , M. Mahmoudi , Chem. Soc. Rev. 2017, 46, 4218. 28585944 10.1039/c6cs00636a PMC5593313

[6] V. Raman , N. Van Dessel , O. M. O'connor , N. S. Forbes , J Immunother Cancer 2019, 7, 44. 30755273 10.1186/s40425-018-0490-z PMC6373116

[7] W. Kong , J. Clark‐Curtiss , R. Curtiss Iii , Expert Rev. Vaccines 2013, 12, 345. 23560914 10.1586/erv.13.7

[8] C. Pan , J. Wu , S. Qing , X. Zhang , L. Zhang , H. Yue , M. Zeng , B. Wang , Z. Yuan , Y. Qiu , H. Ye , D. Wang , X. Liu , P. Sun , B. Liu , E. Feng , X. Gao , L. Zhu , W. Wei , G. Ma , H. Wang , Adv. Mater. 2020, 32, 2002940. 10.1002/adma.202002940 32881121

[9] C. Sivasankar , C. Hewawaduge , J. H. Lee , J Control Release 2023, 357, 404. 37044178 10.1016/j.jconrel.2023.04.015

[10] W. Kong , J. Clark‐Curtiss , R. Curtiss Iii , Expert Rev. Vaccines 2013, 12, 345. 23560914 10.1586/erv.13.7

[11] C. Sivasankar , C. Hewawaduge , J. H. Lee , J Control Release 2023, 357, 404. 37044178 10.1016/j.jconrel.2023.04.015

[12] J. E. Galen , R. Curtiss , Vaccine 2014, 32, 4376. 24370705 10.1016/j.vaccine.2013.12.026 PMC4069233

[13] W. Kong , M. Brovold , B. A. Koeneman , J. Clark‐Curtiss , R. Curtiss , Proc Natl Acad Sci U S A 2012, 109, 19414. 23129620 10.1073/pnas.1217554109 PMC3511069

[14] H. Nariya , M. Inouye , Cell 2008, 132, 55. 18191220 10.1016/j.cell.2007.11.044

[15] M. Suzuki , L. Mao , M. Inouye , Nat. Protoc. 2007, 2, 1802. 17641648 10.1038/nprot.2007.252

[16] T. Li , Y. Weng , X. Ma , B. Tian , S. Dai , Y. Jin , M. Liu , J. Li , J. Yu , Y. Hua , Front Microbiol 2017, 8, 1427. 28798741 10.3389/fmicb.2017.01427 PMC5526972

[17] J. Cho , A. N. Carr , L. Whitworth , B. Johnson , K. S. Wilson , Microbiology (Reading) 2017, 163, 308. 28113053 10.1099/mic.0.000436

[18] R. Hazan , H. Engelberg‐Kulka , Mol. Genet. Genomics 2004, 272, 227. 15316771 10.1007/s00438-004-1048-y

[19] N. Saito , H. Chono , H. Shibata , N. Ageyama , Y. Yasutomi , J. Mineno , Mol Ther Nucleic Acids 2014, 3, e168. 24914931 10.1038/mtna.2014.20 PMC4078760

[20] J. M. Jacobson , J. K. Jadlowsky , S. F. Lacey , J. A. Fraietta , G. Plesa , H. Chono , D. H. Lee , I. Kulikovskaya , C. Bartoszek , F. Chen , L. Tian , A. Dimitri , B. L. Levine , E. A. Veloso , W.‐T. Hwang , C. H. June , Mol. Ther. 2021, 29, 626. 33186691 10.1016/j.ymthe.2020.11.007 PMC7854306

[21] X. Dong , Y. Chao , Y. Zhou , R. Zhou , W. Zhang , V. A. Fischetti , X. Wang , Y. Feng , J. Li , EMBO Mol. Med. 2021, 13, 13810. 10.15252/emmm.202013810 PMC8261479 34137500

[22] D. Limmathurotsakul , Lancet Glob Health 2017, 5, e157.28104185

[23] V. T. L. Huong , H. B. Long , N. V. Kinh , T. T. D. Ngan , V. T. V. Dung , B. Nadjm , H. R. Van Doorn , N. T. Hoa , P. Horby , H. F. L. Wertheim , J Infect 2018, 76, 159. 28970042 10.1016/j.jinf.2017.09.019 PMC5790056

[24] N. M. Susilawathi , N. M. A. Tarini , N. N. D. Fatmawati , P. I. B. Mayura , A. A. A. Suryapraba , M. Subrata , A. A. R. Sudewi , G. N. Mahardika , Emerg Infect Dis 2019, 25, 2235. 31742523 10.3201/eid2512.181709 PMC6874276

[25] M. Segura , Expert Rev. Vaccines 2015, 14, 1587. 26468755 10.1586/14760584.2015.1101349

[26] K.‐J. Hsueh , J.‐W. Lee , S.‐M. Hou , H.‐S. Chen , T.‐C. Chang , C.‐Y. Chu , Transbound Emerg Dis 2014, 61, e35. 23489297 10.1111/tbed.12067

[27] Y.‐A. Li , Z. Ji , X. Wang , S. Wang , H. Shi , Vet Res 2017, 48, 89. 29268787 10.1186/s13567-017-0494-6 PMC5740921

[28] H. Nariya , M. Inouye , Cell 2008, 132, 55. 18191220 10.1016/j.cell.2007.11.044

[29] B. C. M. Ramisetty , B. Natarajan , R. S. Santhosh , Crit. Rev. Microbiol. 2015, 41, 89. 23799870 10.3109/1040841X.2013.804030

[30] S. Wang , Y. Li , G. Scarpellini , W. Kong , H. Shi , C.‐H. Baek , B. Gunn , S.‐Y. Wanda , K. L. Roland , X. Zhang , P. Senechal‐Willis , R. Curtiss , Infect. Immun. 2010, 78, 3969. 20605977 10.1128/IAI.00444-10 PMC2937466

[31] X. Cai , Y.‐H. Chiu , Z. J. Chen , Mol. Cell 2014, 54, 289. 24766893 10.1016/j.molcel.2014.03.040

[32] R. Curtiss , S.‐Y. Wanda , B. M. Gunn , X. Zhang , S. A. Tinge , V. Ananthnarayan , H. Mo , S. Wang , W. Kong , Infect. Immun. 2009, 77, 1071. 19103774 10.1128/IAI.00693-08 PMC2643627

[33] W. Kong , M. Brovold , B. A. Koeneman , J. Clark‐Curtiss , R. Curtiss , Proc. Natl. Acad. Sci. USA 2012, 109, 19414. 23129620 10.1073/pnas.1217554109 PMC3511069

[34] C. R. Beuzon , EMBO J. 2000, 19, 3235. 10880437 10.1093/emboj/19.13.3235 PMC313946

[35] A. Li , M. Yi , S. Qin , Y. Song , Q. Chu , K. Wu , J. Hematol. Oncol. 2019, 12, 35. 30935414 10.1186/s13045-019-0721-x PMC6444510

[36] X. Liu , J. Liu , D. Liu , Y. Han , H. Xu , L. Liu , X. Leng , D. Kong , Biomater. Sci. 2019, 7, 5516. 31670734 10.1039/c9bm01183h

[37] F. Hayashi , K. D. Smith , A. Ozinsky , T. R. Hawn , E. C. Yi , D. R. Goodlett , J. K. Eng , S. Akira , D. M. Underhill , A. Aderem , Nature 2001, 410, 1099. 11323673 10.1038/35074106

[38] J. Von Moltke , J. S. Ayres , E. M. Kofoed , J. Chavarría‐Smith , R. E. Vance , Annu. Rev. Immunol. 2013, 31, 73. 23215645 10.1146/annurev-immunol-032712-095944

[39] F. Nimmerjahn , J. V. Ravetch , Immunity 2006, 24, 19. 16413920 10.1016/j.immuni.2005.11.010

[40] W. Kong , S.‐Y. Wanda , X. Zhang , W. Bollen , S. A. Tinge , K. L. Roland , R. Curtiss , Proc Natl Acad Sci U S A 2008, 105, 9361. 18607005 10.1073/pnas.0803801105 PMC2453710

[41] M. Suzuki , J. Zhang , M. Liu , N. A. Woychik , M. Inouye , Mol. Cell 2005, 18, 253. 15837428 10.1016/j.molcel.2005.03.011

[42] E. D. Brown , E. I. Vivas , C. T. Walsh , R. Kolter , J. Bacteriol. 1995, 177, 4194. 7608103 10.1128/jb.177.14.4194-4197.1995 PMC177162

[43] L. Kang , A. C. Shaw , D. Xu , W. Xia , J. Zhang , J. Deng , H. F. Wo?Ldike , Y. Liu , J. Su , J. Bacteriol. 2011, 193, 1098. 21193606 10.1128/JB.01027-10 PMC3067588

[44] F. V. Marinho , S. Benmerzoug , S. C. Oliveira , B. Ryffel , V. F. J. Quesniaux , Trends Microbiol. 2017, 25, 906. 28625530 10.1016/j.tim.2017.05.008 PMC5650497

[45] S. Van Herck , B. Feng , L. Tang , Adv Drug Deliv Rev 2021, 179, 114020. 34756942 10.1016/j.addr.2021.114020

[46] J. Conlon , D. L. Burdette , S. Sharma , N. Bhat , M. Thompson , Z. Jiang , V. A. K. Rathinam , B. Monks , T. Jin , T. S. Xiao , S. N. Vogel , R. E. Vance , K. A. Fitzgerald , J. Immunol. 2013, 190, 5216. 23585680 10.4049/jimmunol.1300097 PMC3647383

[47] D. J. Patel , Y. Yu , W. Xie , Nat. Struct. Mol. Biol. 2023, 30, 245. 36894694 10.1038/s41594-023-00933-9 PMC11749898

[48] S. Rose‐John , K. Winthrop , L. Calabrese , Nat Rev Rheumatol 2017, 13, 399. 28615731 10.1038/nrrheum.2017.83

[49] G. Trinchieri , Nat. Rev. Immunol. 2003, 3, 133. 12563297 10.1038/nri1001

[50] Y. Xiao , F. Liu , J. Yang , M. Zhong , E. Zhang , Y. Li , D. Zhou , Y. Cao , W. Li , J. Yu , Y. Yang , H. Yan , Cell Mol. Immunol. 2015, 12, 729. 25418468 10.1038/cmi.2014.110 PMC4716618

[51] M. K. Skouboe , A. Knudsen , L. S. Reinert , C. Boularan , T. Lioux , E. Perouzel , M. K. Thomsen , S. R. Paludan , PLoS Pathog. 2018, 14, e1006976. 29608601 10.1371/journal.ppat.1006976 PMC5897032

[52] L. Hatscher , L. Amon , L. Heger , D. Dudziak , Immunol. Lett. 2021, 234, 16. 33848562 10.1016/j.imlet.2021.04.002

[53] J. Wang , P. Li , Y. Yu , Y. Fu , H. Jiang , M. Lu , Z. Sun , S. Jiang , L. Lu , M. X. Wu , Science 2020, 367, eaau810. 10.1126/science.aau0810 PMC7432993 32079747

[54] Y. Wang , B. Jiang , Y. Guo , W. Li , Y. Tian , G. F. Sonnenberg , J. N. Weiser , X. Ni , H. Shen , Mucosal Immunol. 2017, 10, 250. 27118490 10.1038/mi.2016.41 PMC5083242

[55] Y. Luo , C. Li , Z. Zhou , Z. Gong , C. Zhu , A. Lei , Immunology 2021, 164, 223. 33930194 10.1111/imm.13346 PMC8442233

[56] E. Van Dis , K. M. Sogi , C. S. Rae , K. E. Sivick , N. H. Surh , M. L. Leong , D. B. Kanne , K. Metchette , J. J. Leong , J. R. Bruml , V. Chen , K. Heydari , N. Cadieux , T. Evans , S. M. Mcwhirter , T. W. Dubensky , D. A. Portnoy , S. A. Stanley , Cell Rep. 2018, 23, 1435. 29719256 10.1016/j.celrep.2018.04.003 PMC6003617

[57] A. Ziegler , L. Gonzalez , A. Blikslager , Cell Mol. Gastroenterol. Hepatol. 2016, 2, 716. 28090566 10.1016/j.jcmgh.2016.09.003 PMC5235339

[58] K. Roland , R. Curtiss , D. Sizemore , Avian Dis 1999, 43, 429. 10494411

[59] Z. Ji , J. Shang , Y. Li , S. Wang , H. Shi , Vaccine 2015, 33, 4858. 26238722 10.1016/j.vaccine.2015.07.063

[60] H.‐Y. Cheng , V. W. C. Soo , S. Islam , M. J. Mcanulty , M. J. Benedik , T. K. Wood , Environ. Microbiol. 2014, 16, 1741. 24373067 10.1111/1462-2920.12373

[61] X. Zhang , S.‐Y. Wanda , K. Brenneman , W. Kong , X. Zhang , K. Roland , R. Curtiss , BMC Microbiol. 2011, 11, 31. 21303535 10.1186/1471-2180-11-31 PMC3047425

[62] M. Matumoto , Jpn J Exp Med 1949, 20, 175. 15396956

[63] B. Ilyas , D. T. Mulder , D. J. Little , W. Elhenawy , M. M. Banda , D. Pérez‐Morales , C. N. Tsai , N. Y. E. Chau , V. H. Bustamante , B. K. Coombes , Cell Rep. 2018, 25, 825. 30355489 10.1016/j.celrep.2018.09.078

[64] L. Loyal , J. Braun , L. Henze , B. Kruse , M. Dingeldey , U. Reimer , F. Kern , T. Schwarz , M. Mangold , C. Unger , F. Dörfler , S. Kadler , J. Rosowski , K. Gürcan , Z. Uyar‐Aydin , M. Frentsch , F. Kurth , K. Schnatbaum , M. Eckey , S. Hippenstiel , A. Hocke , M. A. Müller , B. Sawitzki , S. Miltenyi , F. Paul , M. A. Mall , H. Wenschuh , S. Voigt , C. Drosten , R. Lauster , et al., Science 2021, 374, eabh1823. 34465633 10.1126/science.abh1823 PMC10026850

[65] Y.‐A. Li , Y. Sun , Y. Fu , Y. Zhang , Q. Li , S. Wang , H. Shi , Vet Res 2022, 53, 46. 35733156 10.1186/s13567-022-01062-9 PMC9215036

