# Supplementary material for: A Bacterial mRNA‐Lysis‐Mediated Cargo Release Vaccine System for Regulated Cytosolic Surveillance and Optimized Antigen Delivery
Source: Adv Sci (Weinh). 2023 Oct 22;10(33):2303568. doi: 10.1002/advs.202303568 (PMC10667801; doi:10.1002/advs.202303568)
Supplement: Supplementary file 1 — Supporting Information [file ADVS-10-2303568-s001.pdf]

ADVANCED SCIENCE
Open Access

Supporting Information

for *Adv. Sci.*, DOI 10.1002/advs.202303568

A Bacterial mRNA-Lysis-Mediated Cargo Release Vaccine System for Regulated Cytosolic Surveillance and Optimized Antigen Delivery

Yu-an Li, Yanni Sun, Yuqin Zhang, Quan Li, Shifeng Wang, Roy Curtiss III and Huoying Shi**

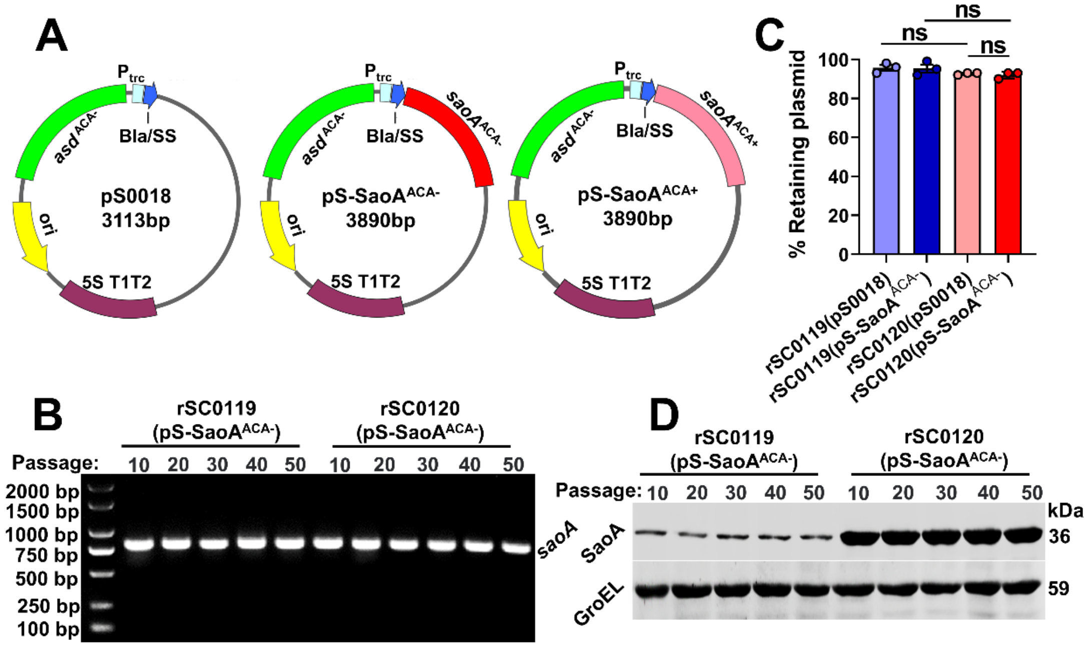

Figure S1. Plasmid stability

(A) Plasmid maps of control vector pS0018, expression vector pS-SaoA^{ACA-}, and pS-SaoA^{ACA+}. ACA sequences in *asd* in pS0018 were removed without altering its amino acid sequence. *SaoA* was engineered to have be ACA-less in pS-SaoA^{ACA-} without altering its amino acid sequence. *SaoA* in pS-SaoA^{ACA+} has ACA sequences. (B) The presence of *saoA* in *S*. Choleraesuis strains with SaoA plasmid was determined by PCR for ~50 generations. (C) Strains were passaged for ~50 generations in a nonselective medium, and the percent plasmid retention of both plasmids was determined by CFU enumeration upon plating on both nonselective medium and selective medium after the final passage. Plasmid stability was determined as the percentage of colonies (out of 100 selected) growing on selective media. Data are expressed as the mean ± SEM. Adjusted *P* values were calculated by one-way ANOVA with Tukey's multiple comparison test. Asterisks indicate significant differences between groups linked by horizontal lines. ns, not significant. (D) The synthesis of SaoA in *S*. Choleraesuis strains

with SaoA plasmid was determined by western blot. GroEL was used as the loading control.

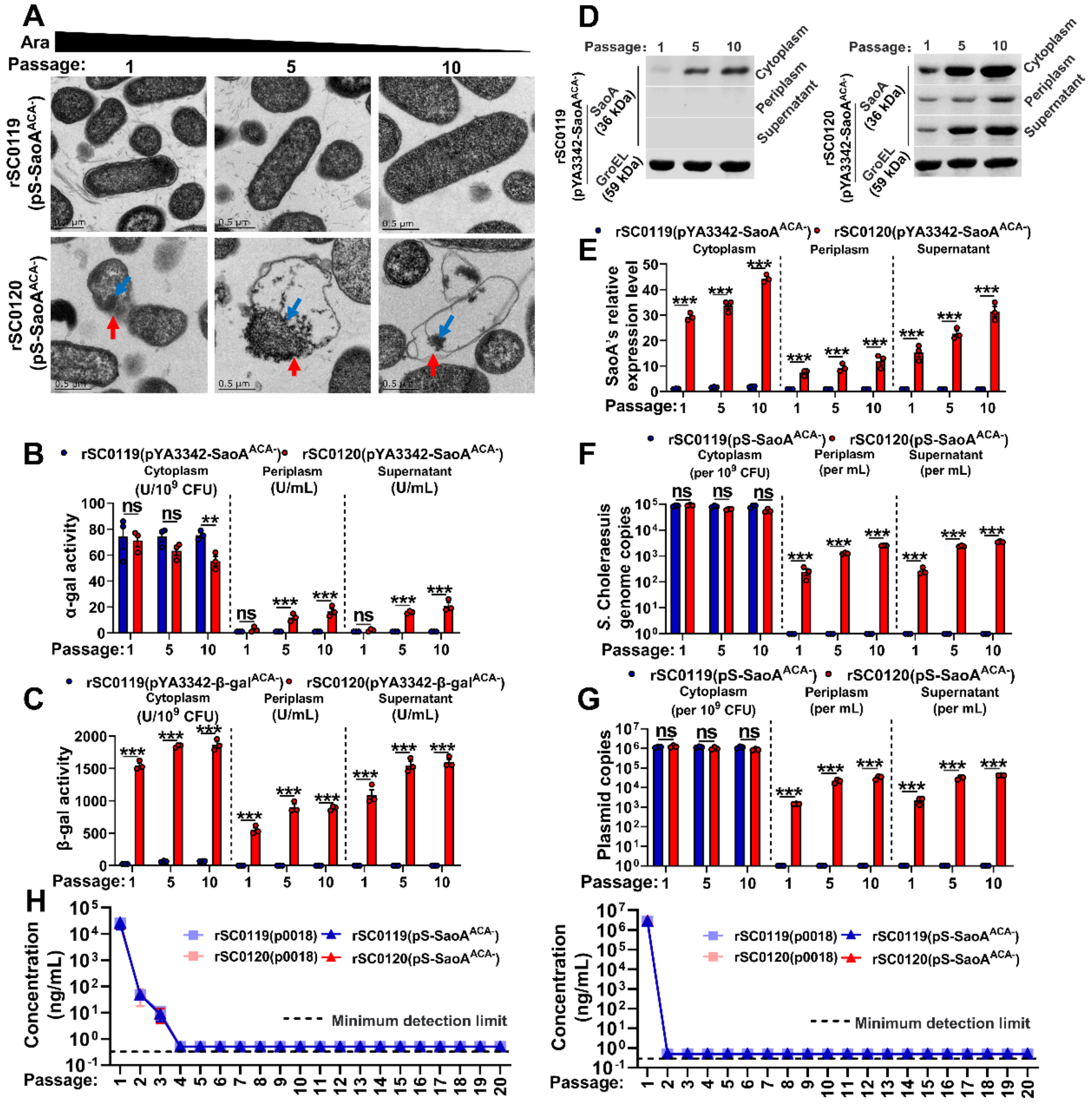

Figure S2. Arabinose regulates self-lysis and cargo release of SIRV

The indicated strains were serially passaged in an arabinose-free medium, supplemented with 0.2% (w/v) arabinose during primary passage. (A) TEM analysis of rSC0119(pS-SaoA^{ACA-}) and rSC0120(pS-SaoA^{ACA-}) at passages 1, 5, and 10. Scale bars

represent 5 μm. Red arrows point to the presence of trans-membrane tunnels on cells, and blue arrows point to the release of cytoplasmic contents. Scale bars represent 0.5 μm. (B) The subcellular localizations of *Salmonella* intrinsic α-gal and (C) foreign β-gal in indicates strains at passages 1, 5, and 10. (D) Chromosomal DNA and (E) plasmid DNA in cytoplasm, periplasm, and supernatant at passages 1, 5, and 10 by qRT-PCR. SaoA production in cytoplasm, periplasm, and supernatant were analysed at passages 1, 5, and 10 with antibody against SaoA. (F) Representative images. (G) Statistical analysis histograms. (H) Bacterial cell ultrasonication samples and supernatant of each passage were collected for HPLC analysis to detect the concentration of arabinose. (A) n = 3 biological replicates per group and a representative sample is showed. (B-H) n = 3 biological replicates per group. Data Are expressed as the mean ± SEM. Adjusted P values were calculated by one-way ANOVA with Tukey's multiple comparison test. Asterisks indicate significant differences between groups linked by horizontal lines. ns, not significant; ***, $P < 0.001$.

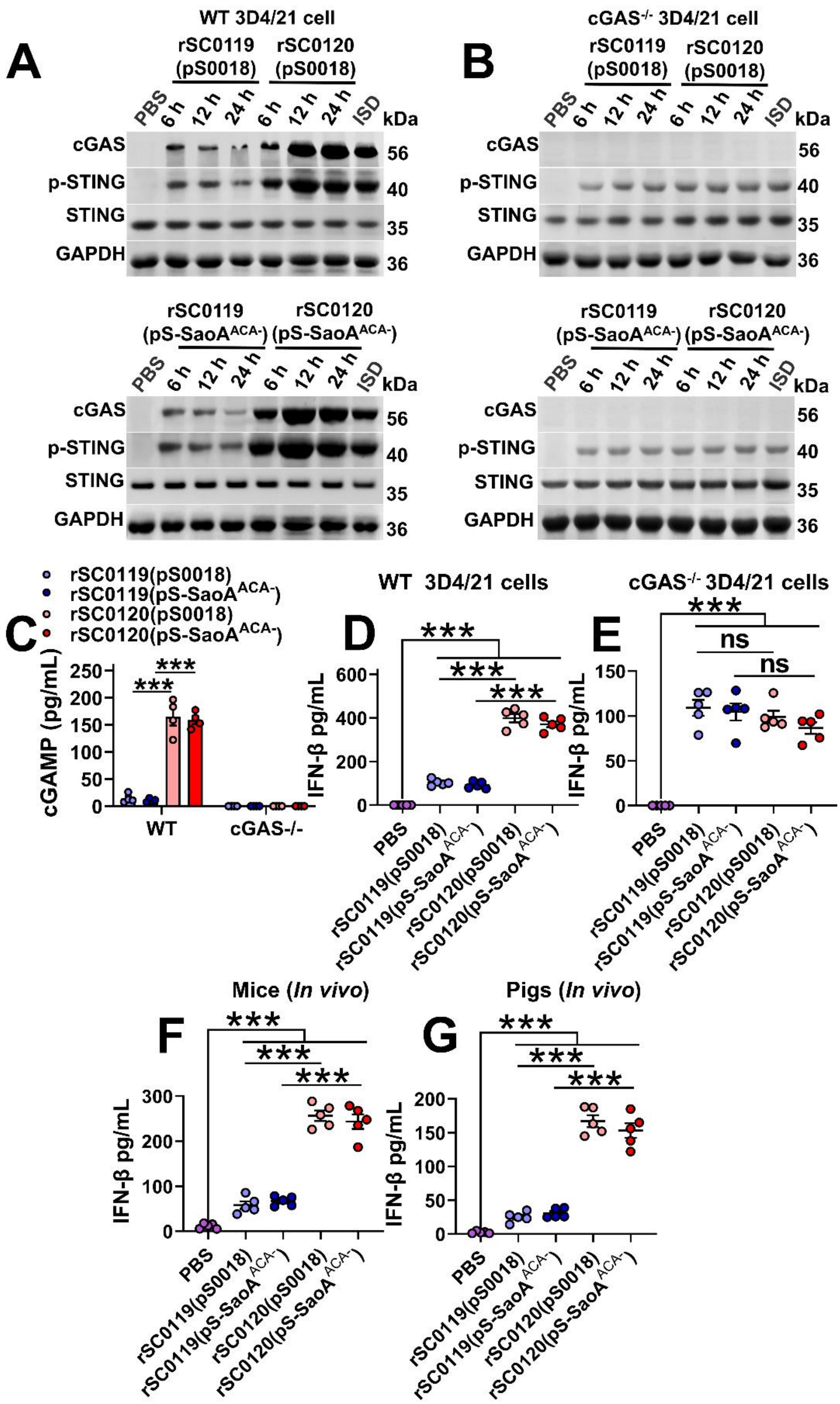

Figure S3. SIRV enhanced the activation of the cGAS-STING axis in 3D4/21 cells and in vivo

At 0 h, 1 h, 6 h, 12 h, and 24 h post-infection with indicated strains, cGAS and p-STING

expression in WT 3D4/21 cells (A) and cGAS KO 3D4/21 cells (B) were assessed by western blot. For all western blots, GAPDH was used as a loading control. (C) Analysis of catalytic activity of cGAS in 3D4/21 cells infected with the relevant strains. The cGAMP in cell extracts was separated by chromatography using a C18 column, and the cGAMP was quantitated by LC-MS analysis. Secretory IFN-β levels in were assessed by ELISA 12 h post-infection in WT 3D4/21 cells (D) and cGAS KO D4/21 cells (E). Secretory IFN-β levels in mice (F) or pigs (G) serum were assessed by ELISA 24 h post-primary immunization. (A, B) $n = 3$ biological replicates per group and a representative sample is showed. (C) $n = 4$ biological replicates per group. (D, E) $n = 5$ biological replicates per group. (F, G) $n = 5$ mice or pigs. (C-G) Data Are expressed as the mean ± SEM. Adjusted P values were calculated by one-way ANOVA with Tukey's multiple comparison test. Asterisks indicate significant differences between groups linked by horizontal lines. ns, not significant; ***, $P < 0.001$; **, $P < 0.01$.

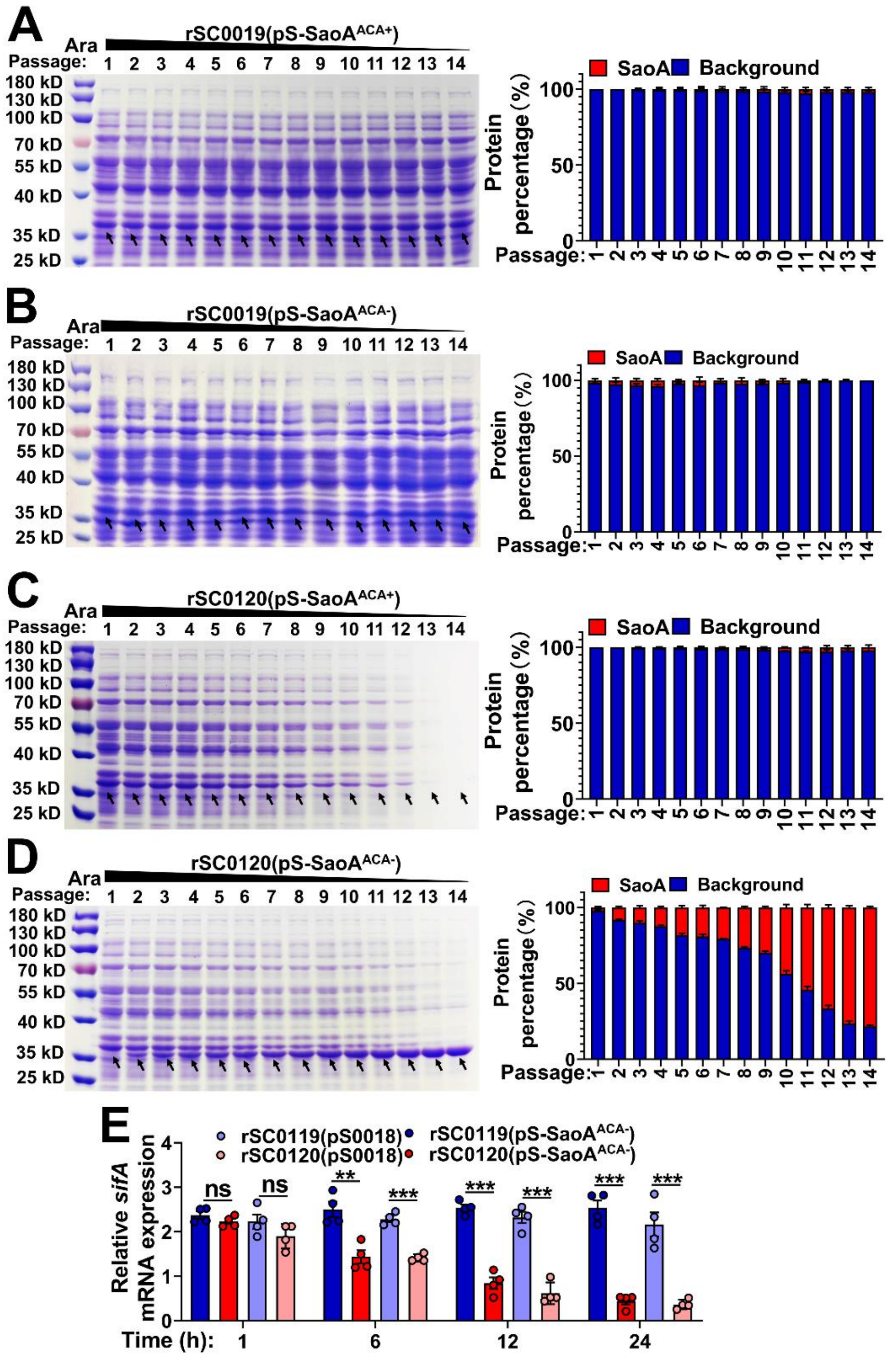

Figure S4. SIRV mediates the enrichment and release of foreign antigens

The rSC0119 or rSC0120 carrying pS-SaoA^{ACA+} (A, C) or pS-SaoA^{ACA-} (B, D) were serially passaged in arabinose-free medium, supplemented with 0.2% (w/v) arabinose during primary generation. SDS-PAGE analysis of the effects of arabinose regulates the expression of target proteins with or without ACA sequences. Black arrows point to

SaoA. Representative images are on the left, and densitometric measurements showing the proportion of SaoA are on the right (mean ± SEM). n = 3 biological replicates per group and a representative sample is showed. (E) SCV-control genes *sifA* measured by qRT-PCR and expressed as fold change in transcript levels relative to GroEL at 1 h, 6 h, 12 h, and 24 h post-infection with indicated strains. n = 4 biological replicates per group. Data are expressed as the mean ± SEM. Adjusted P values were calculated by one-way ANOVA with Tukey's multiple comparison test. Asterisks indicate significant differences between groups linked by horizontal lines. ns, not significant. *** $P < 0.001$.

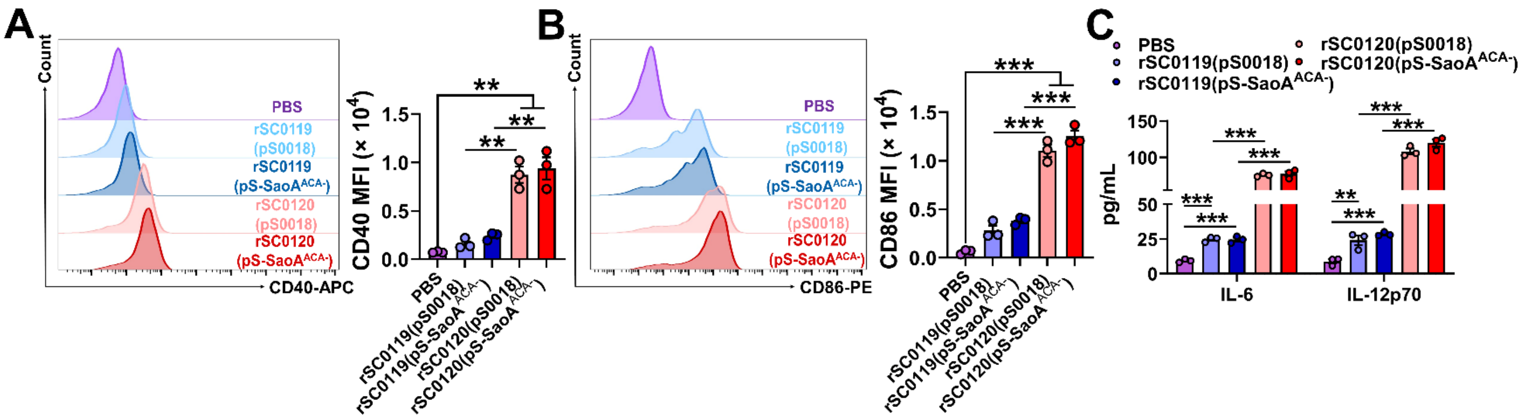

Figure S5. SIRV induces maturation of BMDMs

The mean fluorescence intensity (MFI) of SaoA in BMDMs cytoplasm was quantified by means of flow cytometry. Representative images are on the left, and statistical analysis histograms are on the right. BMDMs were treated with PBS, rSC0119(pS0018), rSC0119(pS-SaoA^{ACA-}), rSC0120(pS0018) or rSC0120(pS-SaoA^{ACA-}). The MFI of CD40 (A) and CD86 (B) on these BMDMs were quantified by means of flow cytometry. Representative images are on the left, and statistical analysis histograms are on the right. (C) Cytokine IL-6 and IL-12p70 levels in BMDMs supernatants, only those cytokines with detectable levels are shown. n = 3 biological replicates per group. Data are

expressed as the mean ± SEM. Adjusted P values were calculated by one-way ANOVA with Tukey's multiple comparison test. Asterisks indicate significant differences between groups linked by horizontal lines. *** $P < 0.001$, ** $P < 0.01$.

Table S1 ACA base triplets distribution in *Salmonella* serotypes

Serotype / (Accession)	Total number of genes	Number of genes containing ACA nucleotide sequence	Percentage of genes containing ACA nucleotide sequence
Salmonella Choleraesuis (AE017220.1)	4704	4544	96%
Salmonella Typhimurium (NC_016863.1)	4662	4453	96%
Salmonella Enteritidis (CP013097.1)	4370	4300	98%
Salmonella Dublin (CP032449.1)	5041	4861	96%
Salmonella Pullorum (NZ_CP012347.1)	4544	4329	95%

Table S2

**Number of ACA nucleotide sequence of investigated genes in *Salmonella* serotypes**		
Serotype / (Accession No.)	Genes/Locus_tag	Number of ACA
Salmonella Choleraesuis (AE017220.1)	*murA*/SCH_3245	11
	alr/SCH_4126	11
	dadX/SCH_1795	12
	asd/SCH_3469	10
	sifA/SCH_1174	17
Salmonella Typhimurium (NC_016863.1)	*murA*/STMUK_3293	11
	alr/STMUK_4232	11
	dadX/STMUK_1774	12
	asd/STMUK_3524	10
	sifA/STMUK_1192	10

Salmonella Enteritidis (CP013097.1)	*murA*/AC092_16245	11
	alr/AC092_20825	12
	dadX/AC092_06370	13
	asd/AC092_21335	10
	sifA/AC092_09455	11
Salmonella Dublin (CP032449.1)	*murA*/DZA56_02920	11
	alr/DZA56_14055	19
	dadX[a]	NA
	asd/DZA56_01730	10
	sifA/DZA56_10930	17
Salmonella Pullorum (NZ_CP012347.1)	*murA*/SEEP9120_RS20425	10
	alr/SEEP9120_RS02450	12
	dadX/SEEP9120_RS25660	13
	asd/SEEP9120_RS01480	7
	sifA/SEEP9120_RS09115	10
Escherichia coli (NZ_CP014272.1)	*mazE*/C3026_RS15265	2
	mazF/C3026_RS15260	9
	lacI/C3026_RS04875	11
**Number of ACA nucleotide sequence of flagellin genes in *S*.Choleraesuis**		
Genes/Locus_tag	Number of ACA	
fliC/SCH_1962	25	
fliB/SCH_1961	12	
fliD/SCH_1964	23	
fliF/SCH_1974	20	
fliH/SCH_1976	11	
fliK/SCH_1979	18	

a. *S*. Dublin does not contain *dadX/B*. Blast the homologue of *dadX* from (which serotype) found homologue in the *alr*.

Table S3 LD50 of SIRV

Strain	Description	LD_{50} via intraperitoneal injection	LD_{50} via oral inoculation
C78-3	Wild type	1.3×10^{2} CFU	6.0×10^{4} CFU
rSC0119(pS-SaoA^{ACA-})	Δ*relA*::*araC* P_{araBAD} *lacI* TT, Δ*manA*, Δ*endA*::*araC* P_{araBAD} *mazE* TT, Δ*asdA*	3.6×10^{5} CFU	1.4×10^{10} CFU
rSC0120(pS-SaoA^{ACA-})	Δ*relA*::*araC* P_{araBAD} *lacI* TT, Δ*manA*, Δ*endA*::TT *araC* P_{araBAD} *mazE* TT, Δ*cysG*:P_{lac} *mazF*, Δ*asdA*	6.1×10^{6} CFU	> 5.2×10^{10} CFU

Table S4 Strains, plasmids, and vectors

Bacterial Strains		
Streptococcus suis serotype 2 Wild type, virulent, CVCC3928	Lab. stock	N/A
Streptococcus suis serotype 7 Wild type, virulent, SH04805	Provided by Professor Huochun Yao	N/A
Streptococcus suis serotype 9 Wild type, virulent, GZ0565	Provided by Professor Huochun Yao	N/A
Streptococcus suis serotype 1/2 Wild type, virulent, 2651	Provided by Professor Huochun Yao	N/A
C78-3 Wild type *S.* Choleraesuis, virulent, CVCC79103	Lab stock	N/A
rSC0114, C78-3 + Δ*relA*::*araC* P_{araBAD} *lacI* TT	This study	N/A
rSC0115, C78-3 + Δ*relA*::*araC* P_{araBAD} *lacI* TT, Δ*endA*::*araC* P_{araBAD} *mazE* TT	This study	N/A
rSC0117, C78-3 + Δ*relA*::*araC* P_{araBAD} *lacI* TT, Δ*endA*::*araC* P_{araBAD} *mazE* TT, Δ*cysG*:P_{lac} *mazF*	This study	N/A
rSC0119, C78-3 + Δ*relA*::*araC* P_{araBAD} *lacI* TT, Δ*manA*, Δ*endA*::*araC* P_{araBAD}	This study	N/A

mazE TT, Δ*asdA*		
rSC0120, C78-3 + Δ*relA*::*araC* P_{araBAD} *lacI* TT, Δ*manA*, Δ*endA*::*araC* P_{araBAD} *mazE* TT, Δ*cysG*:P_{lac} *mazF*, Δ*asdA*	This study	N/A
χ7213	Lab. stock	[58]
BL21	Invitrogen	Cat# EC0114
DH5α	Invitrogen	Cat# 18258012
Plasmids		
pYA3493	Lab. stock	[57]
pS0018	This study	N/A
pS-SaoA^{ACA-}	This study	N/A
pS-SaoA^{ACA+}	This study	N/A
pRE112 *sacB mobRP4 R6K oriV oriT* Cm^{r}	Lab. stock	N/A
pYA3736: pRE112Δ*asdA33*,	Lab. stock	N/A
pS003: pRE112 Δ*relA*::*araC* P_{araBAD} *lacI* TT,	Lab. stock	[57]
pRE112 Δ*endA*::TT *araC* P_{araBAD} *mazE* TT	This study	N/A
pRE112 Δ*cysG*:P_{lac} *mazF*	This study	N/A
LentiCRISPRv2	AddGene	Cat# 52961
LentiCRISPRv2-cGAS	This study	N/A

LentiCRISPRv2-STING	This study	N/A
psPAX2	AddGene	Cat# 12260

Talble S5 Primer or gRNA sequences

Primer name	Sequence (5'-3')
Construction of pRE112 Δ*endA*::TT *araC* P_{araBAD} *mazE* TT	
mazE-EcoRI	GGAATTCATGATCCACAGTAGCGTAAAG
mazE-XhoI	GTCACTCGAGATTACCAGACTTCCTTATC
F-*sprT*	AGCTCGATAGACATTATCCG
R-*sprT*	AGCAAAACGAGCCCGCAACGT
F-*yggJ*	TAACCTACACTAGCGGGA
R-*yggJ*	TGTTCGCAGGCGGCGATGGCG
KpnI-Δ*endA*-F-*sprT*	CCGGGTACCAGCTCGATAGACATTATCCG
BglII-Δ*endA*-*R*-*sprT* XhoI-F-P_{araBAD}	GGGAGATCTAGCAAAACGAGCCCGCAACG T CCTCTCGAGCCAAAAAAACGGGTATGGAG A
XbaI-R-*araC*	TTTGAGCTCTGTTCGCAGGCGGCGATGGC G
Construction of pRE112 Δ*cysG*:P_{lac} *mazF*	
KpnI-F-*nirC*	AATGGTACCATCCTGCCGCAAACCTGGCTC
BglII-R-*nirC*	CCCAGATCTTTAATGGTTGGCTGTAACCTT
BglII-P_{lac}-*mazF*	ACTCATTAGGCACCCCAGGCTTTACACTTT ATGCTTCCGGCTCGTA

XbaI-*mazF*-R	GGGTCTAGACTACCCAATCAGTACGTTAAT
XbaI-F-*yhfL*	GGGTCTAGATAATTAAAATAAAGCCCTGAA
SacI-R-*yhfL*	TTTGAGCTCGGCAGGTCGGTGAGAGTTTTG
Construction of plasmid pS-SaoA^{ACA-}	
SaoA-Smal I	CCCGGGCAACCTGATGGGGGCCAGGC
SaoA-Pst I	CTGCAGCATTGCTTCCTTAGAG
Primer for q-RT PCR	
S. Choleraesuis *sch2895*	Forward: GCATATCCTGGGATAACTC
	Reverse: CATCTGCTGTTGTATA
S. Choleraesuis *ygcA*	Forward: TCGCAAAGCGGGTTCCA
	Reverse: GGTATGGCGCAGAATCAT
S. Choleraesuis *sprT*	Forward: GCAGAGGTCGTGCCGCATG
	Reverse: GACGGTGAGTTGATGTTGCTG
S. Choleraesuis *yggL*	Forward: CACCAAACGTCGAAAAGT
	Reverse: GTCGATAAGACTGTCGAT
S. Choleraesuis *nirC*	Forward: CAGCCACGGTCAAATGTG
	Reverse: TGAAGAACAGTACCGTG
S. Choleraesuis *yhfL*	Forward: GCTATCGTACTATACGC
	Reverse: TAATTTTAGAAATAGA
S. Choleraesuis *sifA*	Forward: CATAAACAGCCGCTT
	Reverse: AAATGGTAAAAGAGGT
S. Choleraesuis	Forward: CTTTGGTCGTAAAATAAGGCG

stn	Reverse: TGCCCAAAGCAGAGAGAT
pS-SaoA^{ACA-}	Forward: CCTGATGGGGGCCAGGC
saoA	Reverse: CTTCTGAAATAGTATACCAG
S. Choleraesuis	Forward: GTGCGGAAATTGAAAGCAAT
murA	Reverse: CGCCTTTCACGCGTTCAATA
S. Choleraesuis	Forward: AAGCGGCAACAGTCGTCAT
alr	Reverse: GCTGCGTGATCCCGCCCGCT
S. Choleraesuis	Forward: GACGCCTGTGCTGGTGGACG
dadX	Reverse: TTACGTTGTCACAAACGGCA
S. Choleraesuis	Forward: CGGCACATAATCCGTGGGCG
asd	Reverse: CTACGCCAACTGGCGCAGCA
Mouse GAPDH	Forward: TGGTNCTGGTATAGAGAC
	Reverse: AGTGTCTCTGGTTTTATGTG
Pig β-actin	Forward: CGTCCACTCCGCCAGCACAG
	Reverse: CTTGCTCTGGGCCTCGTC
Pig IL-4	Forward: GTCTCACCTCCCAACTGATC
	Reverse: ATGCACAGAACAGGTCATGT
Pig IFN-γ	Forward: TAGAAATTTTGAAGAATTG
	Reverse: CACTTTGATGAGTTCACTGA
Pig IL-17A	Forward: ATCCTCGTCCCTGTCACTGC
	Reverse: GGACAGAGTTCATGTGATGA
gRNA sequence for CRISPR-Cas9 knockout	

Target to mouse cGAS	GTCGGGGCGCGCTTCGCGGA
ISD	
Sense strand sequsence	TACAGATCTACTAGTGATCTATGACTGATC TGTACATGATCTACA

Table S6 Key resources table

Reagent or resource	Source or reference	Identifier
Antibodies		
Goat anti-mouse IgG-HRP coupling antibody	Sigma	Cat# 12-349
Goat anti-porcine IgG-HRP	Sigma	Cat# AP166P
Anti-rabbit IgG, HRP-linked antibody	Cell Signaling Technology	Cat# 7074
Goat anti-mouse IgG1	Abcam	Cat# ab97240
Goat anti-mouse IgG2a	Abcam	Cat# ab97245
Mouse anti-pig IgG1-HRP	Bio-Rad	Cat# MCA635
Mouse anti-pig IgG2-HRP	Bio-Rad	Cat# MCA635
Goat anti-mouse IgA-HRP coupling antibody	Abcam	Cat# ab97235
Goat anti-pig IgA-HRP coupling antibody	Abcam	Cat# ab112746
PE mouse anti-Rabbit IgG detector	BD Biosciences	Cat# 558553
Biotinylated anti-mouse IFN-γ specific	BD Biosciences	Cat# 554410

antibody		
Biotinylated anti-mouse IL-4 specific antibody	Biolegend	Cat# 504201
Biotinylated anti-mouse IL-17A specific antibody	Abcam	Cat# ab271235
Anti-mouse IFN-γ antibody	BD Biosciences	Cat# 559065
Anti-mouse IL-4 antibody	BD Biosciences	Cat# 555080
Anti-mouse IL-17A antibody	Invitrogen	Cat# PA5-46947
Anti-cGAS antibody	Proteintech	Cat# 26416-1-AP
Anti-STING antibody	Cell Signaling Technology	Cat# 13647
Phospho-STING (Ser366) antibody	Cell Signaling Technology	Cat# 50907
Phospho-STING (Ser365) antibody	Cell Signaling Technology	Cat# 72971
Anti-BrdU antibody	Sigma	Cat# B8434
Anti-MazE antibody	This study	N/A
Anti-MazF antibody	This study	N/A
Anti-SaoA antibody	[27]	N/A
Anti-LacI antibody	Abcam	Cat# ab33832

Anti-GroEL antibody	BD Biosciences	Cat# ab90522
Anti-FliC antibody	BioLegend	Cat# ab93713
Anti-GAPDH antibody	Abcam	Cat# ab8245
F4/80-PE	BD Biosciences	Cat# 565410
CD11c-FITC	BD Biosciences	Cat# 561045
CD86-PE	BD Biosciences	Cat# 551396
CD40-APC	BD Biosciences	Cat# 558695
$CD4^{+}$T cells isolation kit	Miltenyi	Cat# 130-104-453
Anti-Caspase-1 antibody	Abcam	Cat# ab138483
Chemicals, Peptides, and Recombinant Proteins		
RPMI Medium 1640	GIBCO	Cat# 61870-036
DMEM	GIBCO	Cat# C11965500BT
Todd Hewitt Broth	BD Difco	Cat# 249240
Luria-Bertani medium	Oxoid	Cat# CM0996B
Nutrient Broth	BD Difco	Cat# 231000
InstantBlue®	Abcam	Cat# ab119211
BrdU	Sigma	Cat# 19-160
Caspase inhibitor	Abcam	Cat# ab141421
1-Step™ Ultra TMB-ELISA	Thermo Fisher Scientific	Cat# 34028

TRIzol®	Thermo Fisher Scientific	Cat# 15596026
Fast SYBR Green Master Mix	Thermo Fisher Scientific	Cat# 4385614
SYTOX™ Green Ready Flow™	Invitrogen	Cat# R37168
DAPI	Thermo Fisher Scientific	Cat# 62248
Histopaque®-1077	Sigma-Aldrich	Cat# 10771
BSA	Amresco	Cat# 0332
FBS	GIBCO	Cat# 10099141
His-SaoA protein	This study	N/A
His-MazE protein	This study	N/A
His-MazF protein	This study	N/A
Recombinant mouse GM-CSF	R&D Systems	Cat# 415-ML
Recombinant mouse M-CSF	R&D Systems	Cat# 416-ML
IL-4	Abcam	Cat# ab259406
IL-2	Abcam	Cat# ab259380
Protein-G dyna beads	Roche	Cat# 11243233001
His. Bind$^{@}$ purification kit	Novagen	Cat# 70239-3
Hematoxylin-Eosin (HE) staining kit	Sangon Biotech	Cat# E607318-0200

Mouse IL-6 ELISA set	BD Biosciences	Cat# 555240
Mouse IL-12p70 ELISA set	BD Biosciences	Cat# 555256
Mouse IL-1β ELISA set	BD Biosciences	Cat# 559603
Mouse IL-18 ELISA kit	Abcam	Cat# ab216165
Mouse IFN-β ELISA kit	Abcam	Cat# ab252363
Porcine IFN-β ELISA kit	Abcam	Cat# ab273222
Micro BCA protein kit	Beyotime	Cat# P0012S
α-gal assay kit	Solarbio	Cat# BC2570
β-gal assay kit	Solarbio	Cat# BC2580
Experimental models: cell lines		
Mouse: RAW264.7	ATCC	Cat# TIB-71
HEK293	ATCC	Cat# CRL-11268
Pig: 3D4/21	ATCC	Cat# CRL-2843
Experimental models: organisms/strains		
Mouse: BALB/c	Beijing Vital River Laboratory Animal Co. Ltd.	Stock No: 211
Pig: landrace/white mixed breed pigs	Jiangsu Lihua Animal Husbandry Co., LTD	N/A

Oligonucleotides		
Primers	GenScript	see Table S5
Software and algorithms		
GraphPad prism	GraphPad prism	https://www.graphpad.com/scientific-software/prism
Image-J		NIH https://imagej.nih.gov/ij/
BioRender	Illustration Software	https://www.biorender.com/
